# Somatic Symptoms, Anxiety, and Depression Among College Students in the Czech Republic and Slovakia: A Cross-Sectional Study

**DOI:** 10.3389/fpubh.2022.859107

**Published:** 2022-03-11

**Authors:** Beata Gavurova, Viera Ivankova, Martin Rigelsky, Tawfik Mudarri, Michal Miovsky

**Affiliations:** ^1^Department of Addictology, First Faculty of Medicine, Charles University and General Teaching Hospital in Prague, Prague, Czechia; ^2^Faculty of Mining, Ecology, Process Control and Geotechnologies, Institute of Earth Resources, Technical University of Košice, Košice, Slovakia; ^3^Department of Marketing and International Trade, Faculty of Management and Business, University of Prešov, Prešov, Slovakia

**Keywords:** mental health, disorder, prevalence, socio-demographic determinants, COVID-19, PHQ-15, GAD-7, PHQ-9

## Abstract

Studying in college can be a challenging time for many students, which can affect their mental health. In addition to academic pressure and stressful tasks, another aggravating factor in student life is the ongoing coronavirus disease 2019 (COVID-19) pandemic. The aim of the study was to examine the prevalence of anxiety, depression, and somatic symptoms in Czech and Slovak college students during the COVID-19 pandemic and to evaluate possible socio-demographic determinants of mental health problems. A total of 3,099 respondents participated in this cross-sectional study (Czech Republic: 1,422, Slovakia: 1,677). The analyzes included the Patient Health Questionnaire for somatic symptoms (PHQ-15), the Generalized Anxiety Disorder instrument (GAD-7), and the Patient Health Questionnaire for depression (PHQ-9). Socio-demographic factors were gender, age, family structure, marital status, form of study, degree of study, year of study, field of study, distance between home and college, residence, and housing during the semester. Among Czech students, prevalence of somatic complaints, anxiety and depression was 72.2, 40.3, and 52%, respectively. Among Slovak students, prevalence of somatic complaints, anxiety and depression was 69.5, 34.6, and 47%, respectively. During the COVID-19 pandemic, the most severe mental health problems were identified in a non-negligible part of the sample (Czech Republic: PHQ-15 = 10.1%, GAD-7 = 4.9%, PHQ-9 = 3.4%; Slovakia: PHQ-15 = 7.4%, GAD-7 = 3.5%, PHQ-9 = 2.7%). Regarding the differences between the analyzed countries, a significantly higher score in somatic symptoms, anxiety, and depression was identified in the Czech Republic. Significant differences in mental disorders were found in most socio-demographic characteristics. The main results of the logistic regression analysis revealed that risk factors for mental health disorders in Czech and Slovak students were female gender, younger age, third degree of study, and study of Informatics, Mathematics, Information and Communication Technologies (ICT). Especially in the case of these high-risk groups of students, public policies should consider a response to impending problems. The findings are an appeal for a proactive approach to improving the mental health of students and for the implementation of effective prevention programs, which are more than necessary in the Czech and Slovak college environment.

## Introduction

College students are an important element of society in every country, as they are the future driving force, but also consumers of social and health services. Therefore, it is necessary to pay attention to their healthy development, not only physical but also mental. Poor mental health can prevent them from reaching their potential. The period of college study is characterized by various social, psychological, academic, and lifestyle risk factors that can lead students to experience mental health problems such as anxiety and depression (1). Previous literature has addressed many difficulties in student life, however, the most frequent are academic pressure to succeed, balancing priorities, fear of failure, critical incidents, economic and social problems, bad relationships, or post-graduation plans (1–4).

The ongoing coronavirus disease 2019 (COVID-19) pandemic appears to be another challenging phenomenon for college students (5–10). With the onset of the pandemic, hitherto unknown conditions arose in their lives. In order to reduce the spread of COVID-19, strict measures and interventions were implemented around the world (11). Students had to face sudden changes, physical distancing, but also concerns about their health and the health of their loved ones (12). In addition, students experienced distance education and considerable changes in their study habits, with an evident problem being an impairment in concentration and learning abilities (13). All of these COVID-19-related stressors could lead to multiple consequences that can have a psychological impact on them (14). In this context, frustration caused by loss of daily routine, study disruption, loneliness and estrangement, emotional agony and distress, or uncertainty about both the present and future are strong signals of difficult COVID-19 times for students (15, 16). All of this can mean a huge psychological burden for young people, which has many consequences in their lives (17). In the first place, it is poor academic performance (13, 18), but also dropout (19), low quality of life (20), or suicidal thoughts (21, 22) which are characteristic of college students with poor mental health. It is also well-known that depression is associated with the use of addictive substances among students (23, 24). For instance, problematic drinking is common in depressed students (25, 26). In terms of anxiety, similar consequences can be considered. The higher the anxiety, the higher the nicotine dependence among college students (27). Evidence also shows that students with higher anxiety tend to have lower adherence to sleep hygiene behaviors and experience poorer sleep quality which, in turn, negatively affects their academic engagement (28). In other words, students' anxiety has a negative effect on their academic motivation (29). It is also possible to point out the somatic complaints that can occur in college students, not only during the COVID-19 pandemic (30, 31). Somatic complaints are serious concomitant symptoms of poor mental health and should not be overlooked in research. It is considered a somatic response to mental discomfort, or potentially representative of mental health concerns (32).

All of the above-mentioned findings underline the fact that college students are considered a risk group for the psychosocial long-term consequences of the pandemic (30). For these reasons, the attention of academics and professionals should be focused on young people and the determinants of their poor mental health.

Evidence from one Slovak university clearly shows a 2-fold increase in the prevalence of moderate to severe symptoms of anxiety and depression when comparing the pandemic period in late 2020 and the pre-pandemic period in 2018, with factors such as age, loneliness, having close person infected, perceived stress, and low resilience playing an important role (33, 34). Similar results were found in Czech nationwide cross-sectional surveys conducted during the first and second waves of the COVID-19 pandemic. Thus, the prevalence of anxiety almost doubled between 2017 and 2020, and the prevalence of major depression tripled (35). According to the authors of this Czech study, strong concerns about health or economic consequences of COVID-19 were associated with an increased likelihood of having a mental disorder (35). In the Czech Republic, mental health declined sharply during the first wave and showed no improvement during the second wave of the pandemic (36). These valuable findings indicated that mental health problems pose a serious threat across both populations and the situation has worsened since the onset of the pandemic. At the same time, these findings confirmed the critical situation in both countries during the COVID-19 pandemic; therefore, increased attention needs to be paid to the factors associated with poor psychological outcomes. These studies were the main motivation for the authors of the presented study.

Previous studies have mapped the situation and compared pre-pandemic and pandemic periods in the Czech Republic and Slovakia, however, the socio-demographic background of poor mental health has remained unclear. In this critical situation, it is important to know the main determinants of mental health problems in order to identify vulnerable groups and detect emerging disorders in a timely manner. The presented study provides an in-depth examination of the issue, specifically, a more detailed insight into the socio-demographic factors associated with somatic symptoms, anxiety, and depression during the early COVID-19 pandemic.

Theoretically, this study contributes to the knowledge of young people's mental health. Simultaneously, this study helps professionals and public policy makers better understand the issue and develop more effective strategies to improve the mental health of young people. This problem has long been neglected and overlooked in practice in the Czech Republic and Slovakia, and therefore the study can be a valuable platform for a proactive approach with evidence-based interventions.

## Materials and Methods

The main aim of the study was to examine the prevalence of anxiety, depression, and somatic symptoms in Czech and Slovak college students during the COVID-19 pandemic and to evaluate possible socio-demographic determinants of these mental health problems.

### Data Collection and Respondents

The research included primary data collected in the first half of 2020, thus during the first wave of the COVID-19 pandemic in the Czech Republic and Slovakia. Data collection took place in two phases. In the first phase, an online questionnaire was distributed to Czech and Slovak students in their maternal language, mainly throughout emails addressed to academic authorities (deans, vice-deans), academic staff, and members of the university student council. In addition, the questionnaire was shared on social networks, while organic and paid propagations were applied. In the second phase, emails requesting the sharing of the questionnaire with students were addressed to teachers and lecturers of individual universities and individual fields of study. This step was chosen to help collect data in the planned structure of the research sample.

In general, the ambition was to collect data in accordance with the structure of the surveyed populations in both countries. The properties of the sample were based on two main criteria. The first criterion was an adequate representation of colleges, while the research covered 80% of all Czech and Slovak colleges and universities. The second criterion was an adequate proportion of study fields and a minimum of 30 observations in each study field.

A data cleaning process was performed prior to the analyzes. In this regard, 179 respondents were excluded on the basis of their negative answer to the control questionnaire item (a positive answer was needed to claim that one million has 6 zeros, and a numerical expression was also provided). Subsequently, 27 respondents were excluded on the basis of a system error identified in recording their responses (incomplete data). Finally, 87 respondents (foreign students) were excluded on the basis of their nationality, as the research was focused exclusively on domestic students. A total of 3,099 respondents [Czech Republic (CZ) = 1,422; Slovakia (SK) = 1,677] were included in the final research sample. At this point, it should be noted that in several cases of identification variables, obvious errors were identified (e.g., 1,000 as year of birth). These individual responses were removed and considered as missing data in the used analyzes. The socio-demographic profile of the sample is shown in Table 1.

**Table 1:** Socio-demographic profile of the sample.

	**Variable**	***n***	**%**	**% Without missing**
Gender	Male	955	30.8	30.8
	Female	2,144	69.2	69.2
Age	≤ 20	399	12.9	12.9
	21–25	2,130	68.7	68.8
	26–30	314	10.1	10.1
	≥31	251	8.1	8.1
	Missing	5	0.2	–
Family structure	Complete family (mother and father)	2,379	76.8	76.8
	Incomplete (mother only)	199	6.4	6.4
	Incomplete (father only)	44	1.4	1.4
	Divorced parents (living with mother)	421	13.6	13.6
	Divorced parents (living with father)	44	1.4	1.4
	Living only with siblings	3	0.1	0.1
	Orphan	9	0.3	0.3
Marital status	Single	2,826	91.2	91.2
	Married	234	7.6	7.6
	Divorced	37	1.2	1.2
	Widowed	2	0.1	0.1
Degree of study	1st degree	1,798	58.0	58.0
	2nd degree	808	26.1	26.1
	Combined 1st and 2nd degree	91	2.9	2.9
	3rd degree	402	13.0	13.0
Year of study	1st	1,082	34.9	34.9
	2nd	953	30.8	30.8
	3rd	611	19.7	19.7
	4th	199	6.4	6.4
	5th	212	6.8	6.8
	6th	42	1.4	1.4
Form of study	Full-time	2,591	83.6	83.6
	Part-time	508	16.4	16.4
Field of study	Education	357	11.5	11.5
	Humanities & Arts	179	5.8	5.8
	Social, Economic & Legal Sciences	1,336	43.1	43.1
	Natural Science	123	4.0	4.0
	Design, Technology, Production & Communications	257	8.3	8.3
	Agricultural & Veterinary Sciences	120	3.9	3.9
	Health Service	234	7.6	7.6
	Services (tourism, sports, security, transport, logistics)	309	10.0	10.0
	Informatics, Mathematics, ICT	184	5.9	5.9
Distance between home and college	≤ 20.0 kilometers	861	27.8	27.9
	20.1–50.0 kilometers	675	21.8	21.9
	50.1–100.0 kilometers	773	24.9	25.0
	≥100.1 kilometers	779	25.1	25.2
	Missing	11	0.4	-
Residence	Village	1,280	41.3	41.3
	City with up to 10,000 inhabitants	452	14.6	14.6
	City of 10,001–100,000 inhabitants	984	31.8	31.8
	City of 100,001–1,000,000 inhabitants	288	9.3	9.3
	City with over 1,000,001 inhabitants	95	3.1	3.1
Housing during the semester	Dormitory	945	30.5	30.5
	Sublet	426	13.7	13.7
	With family acquaintances	270	8.7	8.7
	With a friend	70	2.3	2.3
	At home	1,388	44.8	44.8

*n, frequency*.

The first degree of study represents a bachelor's study, which is followed by a master's (or engineering) study as the second degree, and the last third degree represents a doctoral study. The combination of the first and second degree represents a specific form that is characteristic of fields of study, such as medical fields.

### Measures

The research focused on anxiety, depression, and somatic symptoms, which were measured by three screening instruments selected from a study conducted by Kroenke et al. (37). Specifically, somatic symptoms were measured using the Patient Health Questionnaire for somatic complaints (PHQ-15), anxiety was measured using the Generalized Anxiety Disorder instrument (GAD-7), and depressive symptoms were identified using the Patient Health Questionnaire for depression (PHQ-9). The PHQ-15 items offered the following possible answers: not bothered−0, bothered a little−1, bothered a lot−2. The answers to the GAD-7 and PHQ-9 items were as follows: not at all−0, several days−1, more than half the days−2, nearly every day−3. For all the measures, the total score was the sum of the answers coded as above. In this way, the somatic symptoms (PHQ-15) and anxiety (GAD-7) scores ranged as follows: 0–4 none, 5–9 mild, 10–14 moderate, 15 and higher scores indicated severe somatic symptoms/anxiety. The depression score (PHQ-9) could be in the following intervals: 0–4 none, 5–9 mild, 10–14 moderate, 15–19 moderately severe, 20 and higher scores indicated severe depression. Thus, the higher the total score, the more serious the mental problem.

### Statistical Analysis

The analytical processing was carried out in three main steps, which were frequency analysis, descriptive analysis and regression analysis. The analyzes were carried out separately for the Czech Republic and separately for Slovakia in order to point out the specificities of these two countries, which share a common history. Frequency analysis was used to point out the prevalence of mental problems in the analyzed population on the basis of its division into individual intervals according to the above-mentioned severity of selected mental disorders. Descriptive analysis of selected mental health indicators was performed in a secondary classification according to the socio-demographic characteristics that are the focus of this study. The central tendency measures (mean, median) were used to identify gross scores in the analyzed data. The Mann-Whitney U test was used to assess differences between two categories, and the Kruskal-Wallis H test was used to assess differences between three or more categories. To their results, η^2^ was also calculated for a better comparison of the effect size of the identifying socio-demographic characteristics. According to Cohen (38), the results can be seen as follows: small effect size (η^2^ = 0.01), medium effect size (η^2^ = 0.06), and large effect size (η^2^ = 0.14). The main analysis was devoted to the application of multiple logistic regression with a binary dependent variable.


(1) $$\begin{array}{l} \ln\left(\frac{p}{1-p}\right)=\;\beta_{1}X_{i1}+\beta_{2}X_{i2}+\ldots +\beta_{k}X_{ik}\;,\left(-\infty ,\infty \right) \end{array}$$

where *p* is the success probability.

The dependent variables, namely somatic symptoms (PHQ-15), anxiety (GAD-7), and depression (PHQ-9) were adjusted to the dichotomous form (0—no mental health problem, 1—mild and higher severity of a mental health problem). For the purpose of this regression analysis, some socio-demographic characteristics were also adjusted into a dichotomous form.

Statistical processing was performed using SPSS Statistic v. 26 (IBM, Inc., Armonk, NY, US) and visualization was performed using Tableau v. 2021.4 (Tableau Software, LLC, Seattle, WA, US).

## Results

In the Results section, the main findings of the research are divided into two subsections according to the applied analysis. The first subsection is devoted to the results of descriptive and frequency analyzes, which provide a first look at the data as well as the prevalence of mental health disorders among students. The second subsection is devoted to the results of the used logistic regression models, which offer an insight into the socio-demographic factors associated with somatic symptoms, anxiety, and depression during the early COVID-19 pandemic.

### Descriptive and Frequency Analyzes

**Figure 1:**
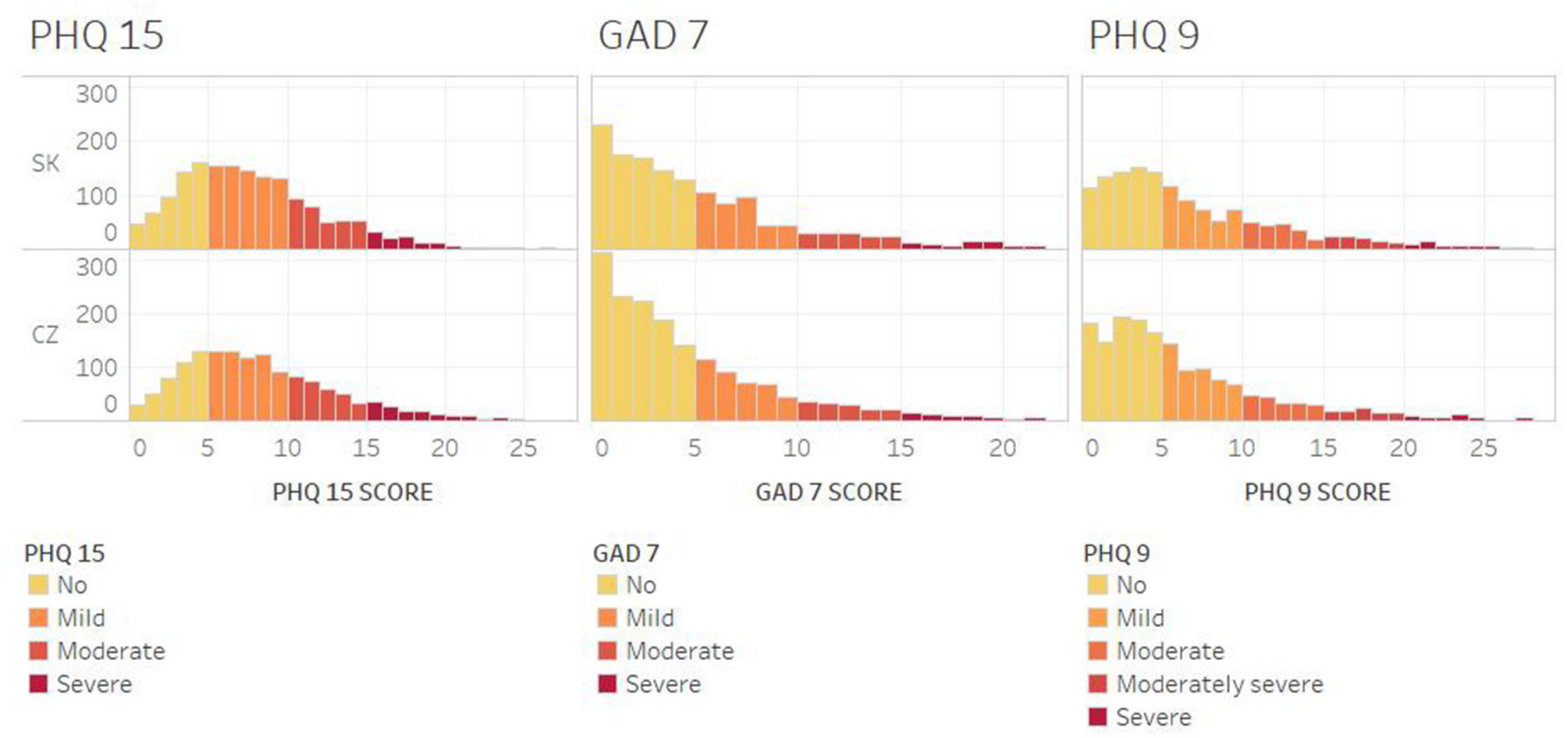
Distribution of PHQ-15, GAD-7 and PHQ-9 scores in the Czech Republic and Slovakia.

Figure 1 shows the distribution of selected mental health problems among Czech and Slovak college students. Overall, students reported the most positive outcomes in anxiety (GAD-7) and, conversely, the least positive outcomes were observed in somatic symptoms (PHQ-15). Similar distributions of selected mental disorders were observed in both countries. Although not obvious in terms of distribution, the results of the Mann-Whitney U test revealed significant differences in all mental disorders (GAD-7: *U* = 1106963.0, *p*-value = 0.001; PHQ-9: *U* = 1113829.5, *p*-value = 0.002; PHQ-15: *U* = 1134734.5, *p*-value = 0.020). Regarding anxiety, a significantly higher GAD-7 score was identified in the Czech Republic (CZ: mean = 4.71 ± 4.6, median = 3; SK: mean = 4.15 ± 4.26, median = 7). Students from the Czech Republic also reported a significantly higher score in the two remaining mental disorders, that is depression (PHQ-9 CZ: mean = 6.34 ± 5.5, median = 5; SK: mean = 5.30 ± 5.30, median = 4) and somatic symptoms (PHQ-15 CZ: mean = 7.77 ± 4.8, median = 7; SK: mean = 7.32 ± 4.6, median = 7). On this basis, it was justified in further analyzes to compare the socio-demographic groups of the population also in the classification of countries.

In the Czech sample, severe somatic symptoms were found in 10.1% of students, severe anxiety in 4.9%, and severe depression in 3.4%. In general, mild and higher rates of mental health problems were identified in 72.2% of students with somatic complaints, 40.3% of students with anxiety, and 52% of students with depression. In the Slovak sample, 7.4% of students reported severe somatic symptoms, 3.5% of students experienced severe anxiety, and 2.7% of students reported severe depression. At the same time, mild and higher rates of mental health problems were found in 69.5% of students with somatic complaints, 34.6% of students with anxiety, and 47% of students with depression.

Table 2 presents the proportion of the most serious rates of mental health problems reported by Czech and Slovak respondents classified according to socio-demographic characteristics. Higher proportion values were observed in several cases; however, COVID-19-related stressors could be reflected in these findings. A more detailed look at the results of the frequency analysis and difference tests is provided in Supplementary Tables 1–9.

**Table 2:** Proportion of severe mental health problems (PHQ-15, GAD-7, PHQ-9) in the classification of selected socio-demographic characteristics—frequency (percentage ratio).

**Variable**	**Category**	**PHQ-15**, ***n*** **(%)**		**GAD-7**, ***n*** **(%)**		**PHQ-9**, ***n*** **(%)**	
		**CZ**	**SK**	**CZ**	**SK**	**CZ**	**SK**
Gender	Male	10 (2.9)	14 (2.3)	6 (1.7)	21 (3.5)	3 (0.9)	19 (3.1)
	Female	134 (12.5)	110 (10.3)	64 (6)	38 (3.5)	46 (4.3)	27 (2.5)
Age	≤ 20	26 (13.5)	10 (4.9)	19 (9.8)	8 (3.9)	17 (8.8)	5 (2.4)
	21–25	95 (10.7)	98 (7.9)	37 (4.2)	42 (3.4)	28 (3.1)	33 (2.7)
	26–30	14 (8.2)	8 (5.6)	11 (6.4)	5 (3.5)	5 (2.9)	5 (3.5)
	≥31.00	9 (5.4)	8 (9.4)	3 (1.8)	3 (3.5)	3 (1.8)	3 (3.5)
Family structure	Complete family	97 (9.5)	100 (7.4)	56 (5.5)	44 (3.2)	36 (3.5)	30 (2.2)
	Incomplete (mother only)	8 (8.7)	8 (7.5)	2 (2.2)	8 (7.5)	3 (3.3)	5 (4.7)
	Incomplete (father only)	2 (8.7)	1 (4.8)	1 (4.3)	2 (9.5)	2 (8.7)	1 (4.8)
	Divorced parents (with mother)	33 (12.9)	14 (8.4)	11 (4.3)	5 (3)	9 (3.5)	9 (5.4)
	Divorced parents (with father)	4 (14.8)	1 (5.9)	0	0	0	0
	Living with siblings, orphan	0	0	0	0	0	1 (14.3)
Marital status	Single	134 (10.7)	118 (7.5)	67 (5.3)	56 (3.6)	47 (3.8)	41 (2.6)
	Married	8 (5.9)	5 (5.1)	2 (1.5)	2 (2)	1 (0.7)	5 (5.1)
	Divorced, widowed	2 (5.9)	1 (20)	1 (2.9)	1 (20)	1 (2.9)	0
Form of study	Full-time	110 (10.6)	115 (7.4)	56 (5.4)	54 (3.5)	41 (3.9)	42 (2.7)
	Part-time	34 (8.9)	9 (7.1)	14 (3.7)	5 (3.9)	8 (2.1)	4 (3.1)
Degree of study	1st degree	68 (10.3)	90 (7.9)	27 (4.1)	42 (3.7)	21 (3.2)	33 (2.9)
	2nd degree	34 (8.9)	23 (5.4)	18 (4.7)	11 (2.6)	8 (2.1)	9 (2.1)
	Combined 1st and 2nd degree	9 (18)	5 (12.2)	7 (14)	3 (7.3)	5 (10)	0
	3rd degree	33 (9.9)	6 (8.8)	18 (5.4)	3 (4.4)	15 (4.5)	4 (5.9)
Year of study	1st	43 (9.1)	45 (7.4)	26 (5.5)	22 (3.6)	21 (4.5)	17 (2.8)
	2nd	43 (9.9)	47 (9.1)	18 (4.1)	16 (3.1)	13 (3)	11 (2.1)
	3rd	48 (15.1)	25 (8.5)	17 (5.3)	14 (4.8)	9 (2.8)	13 (4.4)
	4th	2 (2.1)	2 (1.9)	4 (4.2)	3 (2.9)	2 (2.1)	2 (1.9)
	5th	7 (9.6)	5 (3.6)	5 (6.8)	3 (2.2)	3 (4.1)	2 (1.4)
	6th	1 (3.3)	0	0	1 (8.3)	1 (3.3)	1 (8.3)
Field of study	Education	37 (13.4)	10 (12.5)	18 (6.5)	5 (6.3)	16 (5.8)	6 (7.5)
	Humanities & Arts	8 (7.9)	11 (14.1)	5 (5)	3 (3.8)	2 (2)	4 (5.1)
	Social, Economic & Legal Sciences	60 (9)	38 (5.7)	24 (3.6)	17 (2.5)	14 (2.1)	13 (1.9)
	Natural Science	2 (4)	1 (1.4)	0	2 (2.7)	0	2 (2.7)
	Design, Technology, Production & Communications	5 (5.4)	8 (4.9)	3 (3.2)	9 (5.5)	3 (3.2)	8 (4.9)
	Agricultural & Veterinary Sciences	16 (23.9)	5 (9.4)	14 (20.9)	1 (1.9)	8 (11.9)	1 (1.9)
	Health Service	11 (20.4)	22 (12.2)	4 (7.4)	9 (5)	4 (7.4)	2 (1.1)
	Services (tourism, sports, security, transport, logistics)	3 (4.3)	18 (7.5)	2 (2.9)	6 (2.5)	0	5 (2.1)
	Informatics, Mathematics, ICT	2 (4.3)	11 (8)	0	7 (5.1)	2 (4.3)	5 (3.6)
Distance between home and college	≤ 20.0	48 (10.4)	25 (6.3)	28 (6.1)	20 (5)	20 (4.3)	13 (3.3)
	20.1–50.0	30 (9.4)	17 (4.8)	13 (4.1)	11 (3.1)	9 (2.8)	8 (2.2)
	50.1–100.0	39 (11.2)	32 (7.5)	13 (3.7)	13 (3.1)	10 (2.9)	12 (2.8)
	≥100.1	27 (9.2)	49 (10.1)	16 (5.4)	14 (2.9)	10 (3.4)	12 (2.5)
Residence	Village	39 (8.5)	58 (7)	19 (4.2)	30 (3.6)	13 (2.8)	15 (1.8)
	City (up to 10,000)	19 (7.5)	12 (6.1)	14 (5.5)	7 (3.5)	7 (2.8)	8 (4)
	City (10,001–100,000	58 (12.6)	40 (7.6)	25 (5.4)	16 (3)	21 (4.6)	19 (3.6)
	City (100,001–1,000,000)	15 (8.9)	12 (10.1)	8 (4.7)	5 (4.2)	3 (1.8)	3 (2.5)
	City (over 1,000,001)	13 (15.7)	2 (16.7)	4 (4.8)	1 (8.3)	5 (6)	1 (8.3)
Housing during the semester	Dormitory	26 (10.7)	55 (7.8)	16 (6.6)	18 (2.6)	6 (2.5)	19 (2.7)
	Sublet	28 (9.8)	22 (15.8)	15 (5.2)	9 (6.5)	13 (4.5)	7 (5)
	With family acquaintances	24 (11.9)	6 (8.8)	10 (5)	4 (5.9)	9 (4.5)	4 (5.9)
	With a friend	6 (15)	3 (10)	4 (10)	1 (3.3)	4 (10)	1 (3.3)
	At home	60 (9.2)	38 (5.1)	25 (3.8)	27 (3.7)	17 (2.6)	15 (2)

*PHQ-15, Patient Health Questionnaire for somatic symptoms; GAD-7, Generalized Anxiety Disorder instrument; PHQ-9, Patient Health Questionnaire for depression; n, frequency of severe mental problems; CZ, Czech Republic; SK, Slovakia*.

Supplementary Tables 1–3 show the results for somatic symptoms (PHQ-15) in the classification of selected socio-demographic characteristics of students. In addition to higher rates of somatic complaints, several significant differences in the obtained scores were observed. In both countries, there were significant differences between the gender categories, with higher mean scores for females, and between the age categories, with younger students reporting higher rates of somatic complaints than older students. With a focus on marital status, differences were confirmed at the significance level of α <0.001 in the Czech Republic and at the level of α <0.05 in Slovakia. In terms of the study specifics, both countries showed significant differences between the forms of study, while full-time students were characterized by higher mean scores compared to part-time students. Significant differences were also evident between the fields of study in both countries. In this context, it should be noted that the highest mean score was found for Agricultural & Veterinary Sciences (mean = 10.03) in the Czech Republic. On the contrary, the study of Design, Technology, Production & Communications was characterized by the lowest PHQ-15 score in both countries (mean: CZ = 5.53; SK = 5.90). Significant differences were also identified between the years of the study, but only in Slovakia. In both countries, it was possible to observe significant differences between students' housing during the semester, while students who lived at home during the semester also reported the least somatic complaints (mean: CZ = 7.57; SK = 7.09).

Supplementary Tables 4–6 provide the results for anxiety (GAD-7) in the classification of selected socio-demographic characteristics of students. Again, the difference analysis revealed the most obvious differences in anxiety between the gender categories and between the age categories. Based on the descriptive analysis, female students and younger students acquired higher mean GAD-7 scores in both countries. Significant differences were also identified between the categories of marital status in both countries, with higher mean scores for single students. For the study specifics, the highest score was observed in Czech students with a combined first and second degree (mean = 5.7). However, no significant difference in anxiety was found between the study degrees. On the other hand, both countries showed significant differences between the forms of study, with full-time students reporting higher rates of anxiety than part-time students. In the Czech Republic, there were significant differences between the years of study, as well as between the fields of study. In this context, the highest score was identified in respondents studying Agricultural & Veterinary Sciences (CZ mean = 7.28). Focusing on the years of study, Czech third-year and fifth-year students reported the highest rates of anxiety compared to other years (mean: 3rd = 5.55, 5th = 5.0). In general, it can be stated that no significant differences in anxiety were confirmed between the individual categories of residence. All categories showed approximately similar scores ranging from 2 to 4. A cautious difference at the significance level of α <0.1 was observed for housing during the semester only in Slovakia.

Supplementary Tables 7–9 present the results for depression (PHQ-9) in the classification of selected socio-demographic characteristics of students. As in previous mental disorders, significant differences between the age categories and between the categories of marital status were fully confirmed in both countries. Based on the mean values, it can be noted that younger students suffered from depression more than older students, but also single students reported significantly more depression compared to older students. Gender differences were significant only in the Czech Republic, and female students reported a higher score of depression than male students. In terms of the specifics of the study, significant differences between the forms of study and between the fields of study were confirmed in both countries. In this regard, full-time students acquired higher depression scores than part-time students. Czech respondents studying Agricultural & Veterinary Sciences reported the highest PHQ-9 score (mean = 9.84) compared to others, while respondents studying Design, Technology, Production & Communications reported the lowest mean score (mean = 5.31). In Slovakia, the highest mean score was found in Humanities & Arts (mean = 7.01; median = 5.5), but the median value was higher in the case of Agricultural & Veterinary Sciences (mean = 6.77; median = 6). The lowest mean score was observed in Social, Economic & Legal Sciences (mean = 5.28). Subsequently, significant differences between the degrees of study and between the years of study were revealed only in the Czech Republic. Higher mean scores were evident among Czech students of a combined first and second degree, and among Czech third-year and fifth-year students. In both countries, it was possible to confirm significant differences between students' housing during the semester. Czech students living in dormitories showed the highest depression score (mean = 7.23), while the lowest score was identified for students living at home (mean = 5.9). In Slovakia, students who lived with family acquaintances (mean = 6.63) and in sublet (mean = 6.73) during the semester had the highest depression scores. Similar to the Czech Republic, the lowest depression score was found for students living at home (mean = 5.34). Last but not least, significant differences were found between the distances from home to college in Slovakia, with the highest mean score observed in students traveling more than 100 kilometers (mean = 6.15).

In terms of effect size (η^2^), it can be stated that the highest rates were found in the gender categories for somatic symptoms (PHQ-15), and these rates could be attributed to a medium effect size (η^2^: CZ = 0.108; SK = 0.122). The effect sizes for anxiety (GAD-7) and depression (PHQ-9) were clearly lower, while small effect sizes could be confirmed.

### Logistic Regression Analyzes

This subsection presents the results of the used logistic regression models, the purpose of which was to reveal possible socio-demographic determinants of somatic symptoms, anxiety, and depression among Czech and Slovak college students. As mentioned in the methodology, the dependent variables, namely somatic symptoms (PHQ-15), anxiety (GAD-7), and depression (PHQ-9), were first adjusted to a dichotomous form as follows: 0—no mental health problem, 1—mild and higher severity of a mental health problem. As there were a small number of observations in several socio-demographic categories, some characteristics of students were adjusted to a dichotomous scale (i.e., several categories were merged). This step was to improve the understanding of the results.

Table 3 shows all possible socio-demographic explanatory variables considered in terms of somatic symptoms. It was possible to observe several significant relationships. Being a female increased the probability of somatic symptoms during the early COVID-19 pandemic in both countries. The results revealed that Czech female students were 4.3 times more likely and Slovak female students were 4.23 times more likely to suffer from somatic symptoms. Among Czech students, somatic symptoms were more common in younger individuals, as they had a higher probability of somatic complaints. Specifically, Czech students aged ≤ 25 years (categories: ≤ 20 and 21–25) were more likely to have somatic symptoms than students aged 31 years and over. No significant relationship in terms of age was observed in the Slovak sample. Significant relationships were also found in characteristics related to family status. In this regard, Czech students from an incomplete family were 60% more likely to experience somatic symptoms than Czech students from a complete family, while the other variables remained constant. In terms of the specifics of the study, significant relationships were confirmed in terms of degree of study, years of study, as well as fields of study. Based on the results, it can be concluded that first- and second-degree students were less likely to be somatic than third-degree students only in Slovakia. Focusing on academic years, Czech and Slovak third-year students were approximately 1.5 times more likely to suffer from somatic complaints compared to first-year students. Czech students of Humanities & Arts [odds ratio = 0.4; 95% Confidence Interval (CI) = 0.2–0.78], Social, Economic & Legal Sciences (odds ratio = 0.4; 95% CI = 0.24–0.68), Natural Science (odds ratio = 0.43; 95% CI = 0.19–0.98), and Design, Technology, Production & Communications (odds ratio = 0.36; 95% CI = 0.2–0.66) were less likely to have somatic complaints compared to students of Informatics, Mathematics, Information and Communication Technologies (ICT). In Slovakia, only students of Services (tourism, sports, security, transport, logistics) had a significantly lower probability of somatic symptoms than students of Informatics, Mathematics, ICT. In the category of residence, the results revealed a significant relationship only in the Czech Republic. In this case, students from cities were less likely to be somatic than students from villages (odds ratio = 0.68; 95% CI = 0.52–0.91).

**Table 3:** Logistic regression analysis with somatic symptoms (PHQ-15) as a dependent variable.

**PHQ-15**	**Czech Republic** (Nagelkerke ***R***^**2**^ = 0.383)		**Slovakia** (Nagelkerke ***R***^**2**^ = 0.327)	
	**β (SE) Sig**	**AOR (95% CI)**	**β (SE) Sig**	**AOR (95% CI)**
Gender (reference category: male)				
Female	**1.459 (0.147)** ^**†**^	4.3 (3.22–5.74)	**1.442 (0.126)** ^**†**^	4.23 (3.3–5.41)
Age (reference category: ≥31)				
≤ 20	0.852 (0.366)^**^	2.35 (1.14–4.81)	0.699 (0.418)^*^	2.01 (0.89–4.56)
21–25	0.624 (0.301)^**^	1.87 (1.03–3.37)	0.603 (0.375)	1.83 (0.88–3.82)
26–30	0.375 (0.292)	1.46 (0.82–2.58)	0.36 (0.358)	1.43 (0.71–2.89)
Family structure (reference category: complete)				
Incomplete	0.476 (0.149)^***^	1.61 (1.2–2.15)	0.164 (0.149)	1.18 (0.88–1.58)
Marital status (reference category: not single (married/divorced/widowed)				
Single	0.085 (0.266)	1.09 (0.65–1.83)	0.305 (0.293)	1.36 (0.76–2.41)
Form of study (reference category: part-time)				
Full-time	0.004 (0.207)	1 (0.67–1.51)	−0.051 (0.284)	0.95 (0.54–1.66)
Degree of study (reference category: 3rd degree)				
1st degree	−0.027 (0.162)	0.97 (0.71–1.34)	−0.638 (0.267)^**^	0.53 (0.31–0.89)
2nd degree	0.213 (0.184)	1.24 (0.86–1.77)	−0.686 (0.284)^**^	0.5 (0.29–0.88)
Combined 1st and 2nd	0.026 (0.417)	1.03 (0.45–2.33)	−0.466 (0.482)	0.63 (0.24–1.62)
Year of study (reference category: 1st)				
2nd	0.04 (0.165)	1.04 (0.75–1.44)	0.051 (0.154)	1.05 (0.78–1.42)
3rd	0.388 (0.194)^**^	1.47 (1.01–2.16)	0.403 (0.186)^**^	1.5 (1.04–2.15)
4th	0.405 (0.286)	1.5 (0.86–2.63)	−0.146 (0.265)	0.86 (0.51–1.45)
5th	0.085 (0.312)	1.09 (0.59–2.01)	−0.19 (0.251)	0.83 (0.51–1.35)
6th	−0.656 (0.431)	0.52 (0.22–1.21)	0.217 (0.698)	1.24 (0.32–4.87)
Field of study (reference category: Informatics, Mathematics, ICT)				
Education	−0.311 (0.302)	0.73 (0.41–1.32)	−0.054 (0.337)	0.95 (0.49–1.84)
Humanities & Arts	−0.919 (0.343)^***^	0.4 (0.2–0.78)	0.29 (0.368)	1.34 (0.65–2.75)
Social, Economic & Legal Sciences	**−0.906 (0.265)** ^**†**^	0.4 (0.24–0.68)	−0.378 (0.209)^*^	0.68 (0.45–1.03)
Natural Science	−0.838 (0.418)^**^	0.43 (0.19–0.98)	−0.261 (0.328)	0.77 (0.4–1.46)
Design, Technology, Production & Communications	**−1.023 (0.31)** ^**†**^	0.36 (0.2–0.66)	−0.322 (0.245)	0.72 (0.45–1.17)
Agricultural & Veterinary Sciences	0.023 (0.461)	1.02 (0.41–2.53)	0.656 (0.446)	1.93 (0.8–4.62)
Health Service	−0.329 (0.424)	0.72 (0.31–1.65)	−0.157 (0.278)	0.85 (0.5–1.47)
Services (tourism, sports, security, transport, logistics)	−0.56 (0.373)	0.57 (0.27–1.19)	−0.469 (0.235)^**^	0.63 (0.39–0.99)
Distance between home and college (reference category: ≥100.1)				
≤ 20.0	0.024 (0.19)	1.02 (0.71–1.49)	−0.103 (0.187)	0.9 (0.63–1.3)
20.1–50.0	−0.036 (0.197)	0.96 (0.66–1.42)	0.098 (0.187)	1.1 (0.76–1.59)
50.1–100.0	−0.016 (0.191)	0.98 (0.68–1.43)	−0.048 (0.155)	0.95 (0.7–1.29)
Residence (reference category: Village)				
City	−0.38 (0.144)^***^	0.68 (0.52–0.91)	0.054 (0.117)	1.06 (0.84–1.33)
Housing during the semester (reference category: Home)				
Away from home	0.028 (0.148)	1.03 (0.77–1.37)	−0.05 (0.152)	0.95 (0.71–1.28)

*AOR, adjusted odds ratio (AOR = e^β^); 95% CI, 95% confidence interval*.

* *p-value < 0.1*.

** *p-value < 0.05*.

*** *p-value < 0.01*.

† *p-value < 0.001*.

*Significant results are highlighted in bold*.

Table 4 presents the results of the logistic regression analysis, taking into account all possible socio-demographic explanatory variables in terms of anxiety. The most obvious relationships were found in the categories of gender and degree of study. Anxiety was more common among female students than among male students. Czech females were 1.94 times more prone to anxiety compared to males, while Slovak females had a chance to suffer from anxiety 1.36 times higher than males. With a focus on age, the youngest Czech students aged <20 years were 2 times more likely to be anxious than the oldest students aged 31 years and over. In Slovakia, no significant relationship was found at a significance level of α <0.05. In terms of family status, it can be stated that Czech students from an incomplete family had a 1.3 higher probability of anxiety than students from a complete family. Significant relationships were also observed in the specifics of the study. Czech first- and second-degree students as well as Slovak students of lower than third degree (1st, 2nd, combined) had significantly lower probability of anxiety disorder than doctoral students (third-degree). In the category of the years of study, Slovak fifth-year students were identified with a significantly lower probability of anxiety compared to first-year students (odds ratio = 0.59; 95% CI = 0.35–0.97). In contrast, Czech third-year students were 1.74 times more likely to suffer from anxiety than freshmen. In the category of the study fields, there were several significant relationships with a negative β coefficient. Based on these results, it was possible to conclude that Czech students of Humanities & Arts (odds ratio = 0.4; 95% CI = 0.22–0.73), Social, Economic & Legal Sciences (odds ratio = 0.47; 95% CI = 0.3–0.75), Design, Technology, Production & Communications (odds ratio = 0.34; 95% CI = 0.18–0.62), and Services (odds ratio = 0.47; 95% CI = 0.24–0.91) were identified as significantly less likely to be anxious compared to students of Informatics, Mathematics, ICT. Similar results were observed among Slovak students. Thus, students of Social, Economic & Legal Sciences (odds ratio = 0.57; 95% CI = 0.39–0.84), Natural Science (odds ratio = 0.38; 95% CI = 0.2–0.72), and Services (odds ratio = 0.58; 95% CI = 0.38–0.9) were less likely to have anxiety than students of Informatics, Mathematics, ICT. In terms of the categories related to students' residence, no significant relationship was found.

**Table 4:** Logistic regression analysis with anxiety (GAD-7) as a dependent variable.

**GAD-7**	**Czech Republic** (Nagelkerke ***R***^**2**^ = 0.134)		**Slovakia** (Nagelkerke ***R***^**2**^ = 0.169)	
	**β (SE) Sig**	**AOR (95% CI)**	**β (SE) Sig**	**AOR (95% CI)**
Gender (reference category: male)				
Female	**0.665 (0.145)** ^**†**^	1.94 (1.46–2.58)	0.304 (0.123)^**^	1.36 (1.06–1.72)
Age (reference category: ≥31)				
≤ 20	0.695 (0.327)^**^	2 (1.06–3.81)	0.68 (0.409)^*^	1.97 (0.88–4.4)
21–25	0.32 (0.279)	1.38 (0.8–2.38)	0.721 (0.372)^*^	2.06 (0.99–4.26)
26–30	−0.005 (0.274)	0.99 (0.58–1.7)	0.569 (0.35)	1.77 (0.89–3.51)
Family structure (reference category: complete)				
Incomplete	0.266 (0.125)^**^	1.3 (1.02–1.67)	0.091 (0.135)	1.09 (0.84–1.43)
Marital status (reference category: not single (married/divorced/widowed)				
Single	0.062 (0.245)	1.06 (0.66–1.72)	0.246 (0.285)	1.28 (0.73–2.24)
Form of study (reference category: part-time)				
Full-time	−0.127 (0.183)	0.88 (0.62–1.26)	−0.403 (0.265)	0.67 (0.4–1.12)
Degree of study (reference category: 3rd degree)				
1st degree	**−0.72 (0.143)** ^**†**^	0.49 (0.37–0.64)	**−1.012 (0.242)** ^**†**^	0.36 (0.23–0.58)
2nd degree	−0.367 (0.161)^**^	0.69 (0.51–0.95)	**−0.882 (0.258)** ^**†**^	0.41 (0.25–0.69)
Combined 1st and 2nd	−0.633 (0.331)^*^	0.53 (0.28–1.02)	−1.147 (0.427)^***^	0.32 (0.14–0.73)
Year of study (reference category: 1st)				
2nd	−0.074 (0.148)	0.93 (0.69–1.24)	0.024 (0.142)	1.02 (0.78–1.35)
3rd	0.552 (0.168)^***^	1.74 (1.25–2.42)	0.233 (0.164)	1.26 (0.91–1.74)
4th	−0.331 (0.256)	0.72 (0.43–1.19)	−0.212 (0.253)	0.81 (0.49–1.33)
5th	0.045 (0.278)	1.05 (0.61–1.8)	−0.535 (0.257)^**^	0.59 (0.35–0.97)
6th	−0.533 (0.434)	0.59 (0.25–1.37)	0.002 (0.671)	1 (0.27–3.74)
Field of study (reference category: Informatics, Mathematics, ICT)				
Education	−0.423 (0.259)	0.65 (0.39–1.09)	0.268 (0.298)	1.31 (0.73–2.34)
Humanities & Arts	−0.918 (0.307)^***^	0.4 (0.22–0.73)	−0.255 (0.298)	0.78 (0.43–1.39)
Social, Economic & Legal Sciences	−0.755 (0.235)^***^	0.47 (0.3–0.75)	−0.558 (0.197)^***^	0.57 (0.39–0.84)
Natural Science	−0.561 (0.368)	0.57 (0.28–1.17)	−0.974 (0.328)^***^	0.38 (0.2–0.72)
Design, Technology, Production & Communications	**−1.09 (0.308)** ^**†**^	0.34 (0.18–0.62)	−0.212 (0.237)	0.81 (0.51–1.29)
Agricultural & Veterinary Sciences	−0.461 (0.349)	0.63 (0.32–1.25)	−0.228 (0.333)	0.8 (0.41–1.53)
Health Service	−0.151 (0.355)	0.86 (0.43–1.73)	−0.221 (0.243)	0.8 (0.5–1.29)
Services (tourism, sports, security, transport, logistics)	−0.762 (0.34)^**^	0.47 (0.24–0.91)	−0.539 (0.222)^**^	0.58 (0.38–0.9)
Distance between home and college (reference category: ≥100.1)				
≤ 20.0	−0.152 (0.165)	0.86 (0.62–1.19)	−0.102 (0.173)	0.9 (0.64–1.27)
20.1–50.0	−0.029 (0.172)	0.97 (0.69–1.36)	0.004 (0.17)	1 (0.72–1.4)
50.1–100.0	−0.201 (0.165)	0.82 (0.59–1.13)	−0.116 (0.142)	0.89 (0.67–1.18)
Residence (reference category: Village)				
City	−0.084 (0.123)	0.92 (0.72–1.17)	−0.168 (0.107)	0.84 (0.68–1.04)
Housing during the semester (reference category: Home)				
Away from home	−0.042 (0.13)	0.96 (0.74–1.24)	0.15 (0.14)	1.16 (0.88–1.53)

*AOR, adjusted odds ratio (AOR = e^β^); 95% CI, 95% confidence interval*.

* *p-value < 0.1*.

** *p-value < 0.05*.

*** *p-value < 0.01*.

† *p-value < 0.001*.

*Significant results are highlighted in bold*.

Table 5 shows the results of the logistic regression model with all possible socio-demographic explanatory variables considered in terms of depression. With a focus on gender, Czech female students were 1.53 times more likely to be depressed than male students. However, this was not the case in Slovakia, as no significant relationship was found. Focusing on age, it was possible to confirm that younger students had a higher chance of depression than older students. Compared to the oldest students aged 31 years and over, Czech students aged ≤ 20 years were 3.51 times more likely to suffer from depression, while students aged 21–25 years were 2.36 times more prone to depression. In Slovakia, a significant relationship was found only in the youngest category. In this case, Slovak students aged ≤ 20 years were 2.42 more likely to have depression than students aged ≥31 years. Czech students from an incomplete family were 1.45 times more likely to suffer from depression than students from a complete family. Slovak single students (odds ratio = 1.92; 95% CI = 1.12–3.27) were more likely to be depressed than students with a different marital status (married/divorced/widowed). Regarding the study specifics, Czech students of the first degree were identified with a significantly lower probability of depression compared to students of the third degree (odds ratio = 0.75; 95% CI = 0.57–0.99). At the same time, Slovak students of lower than third degree (1st, 2nd, combined) were less likely to be depressed than doctoral (third-degree) students. Among Czech students, third-year students were more likely to suffer from depression than first-year students. In terms of fields of study, several significant relationships were found in both countries. In these cases, a negative β coefficient indicated a lower probability of depression in students of individual fields of study compared to students of Informatics, Mathematics, ICT. In the Czech Republic, they were students of Humanities & Arts; Social, Economic & Legal Sciences; Natural Science; Design, Technology, Production & Communications; as well as Services. In Slovakia, they were students of Social, Economic & Legal Sciences; Natural Science; Design, Technology, Production & Communications; Health Services; and Services. In the distance category, Slovak students who traveled 50.1–100 kilometers from home to college were 0.75 less likely to be depressed than students traveling more than 100 kilometers.

**Table 5:** Logistic regression analysis with depression (PHQ-9) as a dependent variable.

**PHQ-9**	**Czech Republic** (Nagelkerke R^**2**^ = 0.079)		**Slovakia** (Nagelkerke R^**2**^ = 0.060)	
	**β (SE) Sig**	**AOR (95% CI)**	**β (SE) Sig**	**AOR (95% CI)**
Gender (reference category: male)				
Female	0.427 (0.136)^***^	1.53 (1.17–2)	0.185 (0.115)	1.2 (0.96–1.51)
Age (reference category: ≥31)				
≤ 20	**1.256 (0.324)** ^**†**^	3.51 (1.86–6.62)	0.885 (0.388)^**^	2.42 (1.13–5.18)
21–25	0.86 (0.274)^***^	2.36 (1.38–4.05)	0.65 (0.351)^*^	1.92 (0.96–3.81)
26–30	0.475 (0.267)^*^	1.61 (0.95–2.71)	0.269 (0.335)	1.31 (0.68–2.52)
Family structure (reference category: complete)				
Incomplete	0.37 (0.124)^***^	1.45 (1.14–1.84)	0.021 (0.129)	1.02 (0.79–1.32)
Marital status (reference category: not single (married/divorced/widowed)				
Single	−0.276 (0.241)	0.76 (0.47–1.22)	0.65 (0.273)^**^	1.92 (1.12–3.27)
Form of study (reference category: part-time)				
Full-time	−0.021 (0.178)	0.98 (0.69–1.39)	−0.147 (0.258)	0.86 (0.52–1.43)
Degree of study (reference category: 3rd degree)				
1st degree	−0.288 (0.141)^**^	0.75 (0.57–0.99)	−0.754 (0.242)^***^	0.47 (0.29–0.76)
2nd degree	−0.251 (0.158)	0.78 (0.57–1.06)	−0.792 (0.257)^***^	0.45 (0.27–0.75)
Combined 1st and 2nd	−0.019 (0.341)	0.98 (0.5–1.91)	−1.158 (0.415)^***^	0.31 (0.14–0.71)
Year of study (reference category: 1st)				
2nd	−0.148 (0.144)	0.86 (0.65–1.14)	−0.046 (0.136)	0.96 (0.73–1.25)
3rd	0.4 (0.166)^**^	1.49 (1.08–2.06)	0.281 (0.16)^*^	1.33 (0.97–1.81)
4th	−0.075 (0.24)	0.93 (0.58–1.48)	−0.053 (0.239)	0.95 (0.59–1.52)
5th	0.136 (0.271)	1.15 (0.67–1.95)	−0.104 (0.231)	0.9 (0.57–1.42)
6th	−0.007 (0.401)	0.99 (0.45–2.18)	−0.328 (0.667)	0.72 (0.2–2.66)
Field of study (reference category: Informatics, Mathematics, ICT)				
Education	−0.492 (0.257)^*^	0.61 (0.37–1.01)	−0.165 (0.298)	0.85 (0.47–1.52)
Humanities & Arts	**−1.108 (0.302)** ^**†**^	0.33 (0.18–0.6)	−0.13 (0.297)	0.88 (0.49–1.57)
Social, Economic & Legal Sciences	−0.751 (0.232)^***^	0.47 (0.3–0.74)	**−0.788 (0.195)** ^**†**^	0.45 (0.31–0.67)
Natural Science	−0.854 (0.363)^**^	0.43 (0.21–0.87)	−0.659 (0.295)^**^	0.52 (0.29–0.92)
Design, Technology, Production & Communications	−0.72 (0.285)^**^	0.49 (0.28–0.85)	−0.462 (0.234)^**^	0.63 (0.4–1)
Agricultural & Veterinary Sciences	−0.355 (0.354)	0.7 (0.35–1.4)	−0.315 (0.333)	0.73 (0.38–1.4)
Health Service	−0.209 (0.36)	0.81 (0.4–1.64)	−0.562 (0.242)^**^	0.57 (0.35–0.92)
Services (tourism, sports, security, transport, logistics)	−0.656 (0.328)^**^	0.52 (0.27–0.99)	**−0.831 (0.218)** ^**†**^	0.44 (0.28–0.67)
Distance between home and college (reference category: ≥100.1)				
≤ 20.0	−0.167 (0.162)	0.85 (0.62–1.16)	0.029 (0.166)	1.03 (0.74–1.42)
20.1–50.0	−0.067 (0.169)	0.94 (0.67–1.3)	0.03 (0.164)	1.03 (0.75–1.42)
50.1–100.0	−0.042 (0.162)	0.96 (0.7–1.32)	−0.289 (0.137)^**^	0.75 (0.57–0.98)
Residence (reference category: Village)
City	0.071 (0.121)	1.07 (0.85–1.36)	0.076 (0.103)	1.08 (0.88–1.32)
Housing during the semester (reference category: Home)				
Away from home	0.011 (0.127)	1.01 (0.79–1.3)	0.078 (0.133)	1.08 (0.83–1.4)

*AOR, adjusted odds ratio (AOR = e^β^); 95% CI, 95% confidence interval*.

* *p-value < 0.1*.

** *p-value < 0.05*.

*** *p-value < 0.01*.

† *p-value < 0.001*.

*Significant results are highlighted in bold*.

## Discussion

### Prevalence and Differences in Mental Health Problems

Among Czech students, prevalence of somatic complaints, anxiety and depression was 72.2, 40.3, and 52%, respectively. The most severe mental health problems were found in 10.1% of students with somatic symptoms, in 4.9% of students with anxiety, and in 3.4% of students with depression. Among Slovak students, prevalence of somatic complaints, anxiety and depression was 69.5, 34.6, and 47%, respectively. The highest severity was found in 7.4% of students with somatic symptoms, in 3.5% of students with anxiety, and in 2.7% of students with depression. Hajduk et al. (33) found a higher prevalence of depression and anxiety among Slovak students, but their research took place in December 2020, i.e., during the second wave, when the situation was more critical. In both their studies, Slovak students reported more depression than anxiety (33, 34), which corresponds to the results of the presented study. A very similar prevalence of mental health problems such as anxiety and depression was found among college students from Saudi Arabia (39) and South Korea (40), while students from Brazil showed a higher prevalence of both depression and anxiety (41). In comparison with the results of this study, Duan et al. (42) revealed a higher prevalence of depression but a lower prevalence of anxiety among Chinese college students. Portuguese and Lithuanian students reported a similar prevalence of anxiety but a lower prevalence of depression (43, 44). A slightly lower prevalence of mental disorders was found in a study involving Poland, Slovenia, Ukraine, Russia Germany, Turkey, Israel, and Colombia (9). Thus, the prevalence of mental disorders was similar to other countries (45).

Regarding the differences between the analyzed countries, a significantly higher score in somatic symptoms, anxiety, and depression was identified in the Czech Republic. Significant differences in the obtained scores were also observed in several individual cases, separately for the Czech Republic and Slovakia. Among others, the most obvious differences in mental disorders were found between the gender categories and between the age categories. In this context, it can be stated that female students suffer from mental health problems more than male students, but also younger students reported more mental health problems than older students. There were also other significant differences, especially between the categories of marital status, the categories of study form, the categories of study field, and the categories of housing during the semester.

### Gender Factor

The main findings showed that female gender can be considered as one of the risk factors associated with an increased probability of somatic complaints and anxiety in both countries, and depression in the Czech Republic. This is in line with other studies focusing on students' mental health (9, 14, 39, 46–49). It is a well-known fact that females are more prone to mental disorders and report more mental health problems compared to their male counterparts (50). This can be explained by a lower threshold for perceiving mental impairment in males (51). In addition, the causes of mental problems are more prevalent in females; therefore, females are more likely to develop risk factors for mental disorders than males as early as adolescence (32, 52–54). This can result in more frequent emotional outlets in females (51), but also more frequent symptomatology associated with pain, fatigue, digestive problems, psychomotor agitation, and others (55). All this indicates that females feel and experience difficulties more internally, while biological factors also play an important role (56).

### Age Factor

It was also found that Czech students aged 25 years and under were more likely to have somatic symptoms and depression compared to students aged 31 years and over. At the same time, Czech students aged 20 years and under were more likely to be anxious than students aged 31 years and over. In Slovakia, younger age was found to be a significant factor only in the case of depression. In more detail, Slovak students aged 20 years and under were more likely to suffer from depression than the oldest students (aged 31 years and over). These results agree with the general knowledge that younger people are a vulnerable population group in terms of poor mental health, as evidenced by many authors (39, 46, 57–60). On the other hand, there is also confrontational evidence that older age can be a risk factor in some cases (14). A Hungarian study showed that the younger age of college students can be considered an explanatory variable of favorable mental wellbeing during the COVID-19 isolation (61). Thus, inconsistencies can be observed across studies in different countries. However, the fact remains that special attention should be paid to younger people and their mental health in public policies.

### Family Factor

The fact that college students from an incomplete family more often suffered from mental health problems, such as somatic symptoms, anxiety, and depression, was proven only in the Czech Republic. Despite the fact that this fact did not manifest itself in Slovakia, it is possible to agree with O'Farrell et al. (62), who also found that being from a single-parent family was independently associated with a high depression score. Moreover, a recent study confirmed that being from an incomplete family was associated with a higher lifetime prevalence of major depressive disorder (63). Thus, a family structure is an important determinant of students' mental health not only during the COVID-19 pandemic (64, 65). It is well-known that the family has an irreplaceable place in students' lives, while the presence of both parents is an essential aspect of cohesion, stability and support (66). Gray et al. (67) also emphasized that students reporting sufficient time spent with family members and highest level of love and connectedness, as well as those living in a two-parent family, were happiest. This underlines the importance of the role of parents in students' lives.

This study revealed that being a single student increases the chance of depression only among Slovak students. In other words, single Slovak students were more likely to be depressed than students of a different marital status (married/divorced/widowed). A similar finding was presented by AlHadi and Alhuwaydi (39), who considered a single status to be a main risk factor for anxiety and depression. However, there are also conflicting findings that suggest that married students may be at greater risk of mental discomfort (14, 58). In this study, no significant relationships were confirmed in terms Czech students, as well as mental disorders such as anxiety and somatic complaints. Therefore, this should also be further examined in terms of having a partner.

### Study Specifics

This study did not show that form of study can be considered a determinant of mental discomfort among Czech and Slovak college students. Thus, Czech and Slovak full-time students were not more prone to mental problems compared to part-time students. These findings are inconsistent with those of Stallman (49). According to some authors, full-time students were more negatively impacted by the COVID-19 pandemic, which was reflected in their emotional life (68, 69). On the other hand, Esmaeelzadeh et al. (70) found that part-time students were at higher risk of depression and anxiety than full-time. The form of study did not prove to be significant in the presented research and this fact may reflect the conditions of higher education in the Czech Republic and Slovakia. In any case, these discrepancies with other studies can be followed up with further research.

The findings of this study indicated that Slovak students of lower than third degree (1st, 2nd, combined) had significantly lower probability of anxiety and depression than doctoral (third-degree) students. At the same time, Slovak first- and second-degree students were less likely to be somatic than third-degree students. Among Czech students, first-degree students were less likely to have anxiety and depression and second-degree students were less likely to have anxiety compared to third-degree students. These findings indicate that doctoral students can be considered a risk group (31). The truth is that the degree of study should not be underestimated when examining students' mental health. In this regard, Aristovnik et al. (68) examined the issue from a global perspective and emphasized that first-degree students were generally affected more by the COVID-19 pandemic in terms of their emotional life. Ochnik et al. (9) also revealed that study degree can be a predictor of mental health. In their study focusing on nine countries, it was found that the first degree of study is a risk factor for depression. It is clear that the presented study provided different findings than international and global studies.

With a focus on the years of study, it was found that Czech third-year students were more likely to be anxious, depressed and somatic than first-year students. Slovak third-year students were also more likely to have somatic complaints, but fifth-year students were less likely to have anxiety compared to first-year students. Other studies have also shown that academic years play an important role in students' mental health. In this regard, AlJhani et al. (14) confirmed that first-year students from Saudi Arabia had higher levels of anxiety and stress. Al Saadi et al. (71) found that anxiety was less likely in fifth-and sixth-year compared to second-year students. In other studies, similar findings were revealed in terms of depression, anxiety and stress in other studies (72, 73). Accordingly, it can be agreed that the year of study is one of the main predictors of mental health (4, 47), although the findings may be different, as shown in this study.

The results revealed that the study of Informatics, Mathematics, ICT can be considered a risk factor for mental problems such as somatic symptoms, anxiety and depression in Czech and Slovak students. In other words, the results showed a lower probability of mental problems in students of study fields other than Informatics, Mathematics, ICT. In terms of comparison with other studies, Lipson et al. (74) confirmed that students of Humanities & Art and Design were more likely to have mental health problems. In a study conducted by Odriozola-González et al. (73), students of Humanities & Arts and Social Sciences & Law reported higher scores related to anxiety, depression, and stress with respect to students of Engineering & Architecture. However, their findings are inconsistent with those of Posselt and Lipson (75). It is evident that the field of study should be considered in the mental health of students, as each field is characterized by a different level of difficulty, which may be more pronounced during the COVID-19 pandemic. Academic demands are many times the most obvious aspect of the field of study.

In terms of other characteristics analyzed in this study, the distance between home and college was significant only in Slovak one case, specifically in depression.

### Residence Specifics

Housing during the semester did not appear to be an important determinant of mental problems among Czech and Slovak students. On the other hand, it is possible to point out the findings of Thériault et al. (76), which showed that students living on campus had higher self-efficacy, especially on the subscale of psychological wellbeing, followed by students living off campus with their parents. Students living off campus without their parents had the lowest scores. One significant relationship was also found in the category of residence. In this context, Czech students from cities were less likely to have somatic symptoms than students from villages. Yang et al. (77) also confirmed that rural students had more mental health problems than urban students. At the same time, Zhang et al. (78) pointed to the fact that urban students have significantly higher self-esteem scores than their rural counterparts, but no statistically significant difference in depression was observed between urban and rural students. In contrast, Ochnik et al. (9) analyzed nine countries, including the Czech Republic, and revealed that living in town is a risk factor for depression.

### Implications for Public Health

The level of mental disorders among Czech and Slovak college students was high during the early pandemic. Therefore, the study highlights the importance of monitoring the mental health of college students, communicating problems and developing effective prevention programs. Czech and Slovak colleges should pay increased attention to the mental health of their students and, together with experts and government officials, create mental health policies for successful prevention, early detection and effective treatment of students' mental health problems. In this context, interventions aimed at students' mental health literacy and stigma reduction are necessary (79, 80). Student-centered programs and measures should focus on developing positive coping skills and reducing negative coping behaviors (57, 81).

In addition, barriers to seeking help from mental health professionals should be carefully identified and removed in order to support students' efforts to seek help and to provide timely psychological services with respect to the ongoing pandemic. This study encourages the apparent need for accessible and full-time psychological services in Czech and Slovak colleges to deliver psychological interventions to vulnerable students. College counseling centers play an important role in this regard and have great potential to provide students with professional assistance in improving their mental health (82). Also, electronic counseling centers, digital help-seeking tools and Internet-based interventions have unique features that can make them a key source of support for young people's mental health in modern times, as they are more available and less stressful (83, 84). These tools can provide valuable information, promotional images and videos, online lectures with experts aimed at recognizing the importance of good mental health for young people.

In view of the presented finding, students' individual characteristics such as gender, age, degree of study and field of study should be of great importance when developing mental health programs in the Czech Republic and Slovakia. In this context, female students, younger students, third-degree students, and students of Informatics, Mathematics, ICT were most at risk of mental disorders in both countries. These vulnerable groups of students need special attention and targeted interventions. Nevertheless, the supportive educational interventions should be focused on the college environment as a whole. Family structure and year of study should not be overlooked when developing effective strategies to improve students' mental health. Mental health policies need to focus on health promotion and preventive measures, as the demand for them increases even more during the COVID-19 pandemic. There are many ways to improve students' mental health and achieve their potential in a society, in which education for an active and healthy lifestyle, social and family support, as well as professional adequate help for students with mental health problems are irreplaceable (85). In the case of mental disorders, it is also necessary to be vigilant in terms of substance use (86, 87).

### Strengths, Limitations, and Future Direction

The study has many strengths such as in-depth insight into the problem through many socio-demographic factors, sample size, direct comparison of the Czech Republic and Slovakia, but also coverage of two European countries where insufficient attention was paid to the issue. However, the study did not avoid limitations. Possible limitations include the fact that there was some risk of skewing the results due to non-random sampling. However, the selection of the research sample (quota sampling) was the most suitable alternative in the given conditions of the COVID-19 pandemic. Random selection could not be performed. Nevertheless, this limitation need not be considered disruptive to the results and value of knowledge. Another limitation may be the different measures related to COVID-19 applied in both countries, which may have affected Czech and Slovak students in different ways. Future research ambitions will focus on comparing the results of the pandemic period with the post-pandemic period.

## Conclusions

Good mental health of students should be a priority for college representatives, society, professionals and policy makers, not only during the pandemic period. The study enriches the knowledge base about students' mental health in the Czech Republic and Slovakia. Thus, the main aim of the study was to examine the prevalence of anxiety, depression, and somatic symptoms in Czech and Slovak college students during the COVID-19 pandemic and to evaluate possible socio-demographic determinants of these mental health problems. The results revealed a high prevalence of mental disorders among Czech and Slovak college students and identified vulnerable groups of students, who require a special attention. In this context, female gender, younger age, third-degree (doctoral) study, and study of Informatics, Mathematics, ICT were associated with a higher probability of mental health problems during the early COVID-19 pandemic in both countries. In addition, strategies and interventions aimed at improving students' mental health should also take into account family structure, degree of study, and year of study. The findings of the study can help in efforts to improve students' mental health and implement effective prevention programs, which are more than necessary in both countries.

## Data Availability Statement

The raw data supporting the conclusions of this article will be made available by the authors, without undue reservation.

## Ethics Statement

The research was approved by the Ethics Committee of the General University Hospital in Prague as individual research (Ref. 915/20 S–IV). The study was conducted according to the guidelines of the Declaration of Helsinki. The respondents provided their written informed consent to participate in this study.

## Author Contributions

BG: conceptualization, writing—original draft preparation, writing—review and editing, visualization, supervision, project administration, and funding acquisition. VI: conceptualization, methodology, investigation, resources, writing—original draft preparation, writing—review and editing, visualization, and supervision. MR: conceptualization, methodology, software, data curation, formal analysis, investigation, writing—original draft preparation, and writing—review and editing. TM: resources, writing—original draft preparation, writing—review and editing, visualization, supervision, project administration, and funding acquisition. MM: conceptualization, investigation, writing—original draft preparation, writing—review and editing, supervision, project administration, and funding acquisition. All authors contributed to manuscript revision, read, and approved the submitted version.

## Funding

This research was funded by the Scientific Grant Agency of the Ministry of Education, Science, Research, and Sport of the Slovak Republic and the Slovak Academy Sciences as part of the research project VEGA 1/0797/20: Quantification of Environmental Burden Impacts of the Slovak Regions on Health, Social and Economic System of the Slovak Republic. This study was supported by Institutional Research Program PROGRES No. Q06/LF1.

## Conflict of Interest

The authors declare that the research was conducted in the absence of any commercial or financial relationships that could be construed as a potential conflict of interest.

## Publisher's Note

All claims expressed in this article are solely those of the authors and do not necessarily represent those of their affiliated organizations, or those of the publisher, the editors and the reviewers. Any product that may be evaluated in this article, or claim that may be made by its manufacturer, is not guaranteed or endorsed by the publisher.
