# Supplementary material for: Somatic Symptoms, Anxiety, and Depression Among College Students in the Czech Republic and Slovakia: A Cross-Sectional Study
Source: Front Public Health. 2022 Mar 11;10:859107. doi: 10.3389/fpubh.2022.859107 (PMC8961809; doi:10.3389/fpubh.2022.859107)
Supplement: Supplementary file 1 [file Table_1.docx]

Supplementary Material

**Supplementary Table 1.** Somatic symptoms (PHQ-15) in the categorization of demographic characteristics

| PHQ-15 | | | No  n (%) | Mild  n (%) | Moderate  n (%) | Severe  n (%) | Mean | Median | Statistic (p-value)  η^2^ |
| --- | --- | --- | --- | --- | --- | --- | --- | --- | --- |
| Gender | CZ | Male | 181 (51.9) | 133 (38.1) | 25 (7.2) | 10 (2.9) | 5.00 | 4 | 97270 (<0.001)  0.108 |
|  |  | Female | 215 (20) | 455 (42.4) | 269 (25.1) | 134 (12.5) | 8.67 | 8 |  |
|  | SK | Male | 303 (50) | 235 (38.8) | 54 (8.9) | 14 (2.3) | 5.21 | 4.5 | 178644 (<0.001)  0.122 |
|  |  | Female | 207 (19.3) | 481 (44.9) | 273 (25.5) | 110 (10.3) | 8.52 | 8 |  |
| Age | CZ | ≤20 | 39 (20.2) | 81 (42) | 47 (24.4) | 26 (13.5) | 8.77 | 8 | 32.93 (<0.001)  0.021 |
|  |  | 21–25 | 232 (26) | 373 (41.9) | 191 (21.4) | 95 (10.7) | 7.92 | 7 |  |
|  |  | 26–30 | 57 (33.3) | 64 (37.4) | 36 (21.1) | 14 (8.2) | 7.45 | 7 |  |
|  |  | ≥31 | 68 (41) | 69 (41.6) | 20 (12) | 9 (5.4) | 6.13 | 5 |  |
|  | SK | ≤20 | 58 (28.2) | 83 (40.3) | 55 (26.7) | 10 (4.9) | 7.53 | 7 | 12.82 (0.005)  0.003 |
|  |  | 21–25 | 363 (29.3) | 547 (44.1) | 231 (18.6) | 98 (7.9) | 7.39 | 7 |  |
|  |  | 26–30 | 48 (33.6) | 58 (40.6) | 29 (20.3) | 8 (5.6) | 7.01 | 6 |  |
|  |  | ≥31 | 41 (48.2) | 27 (31.8) | 9 (10.6) | 8 (9.4) | 6.25 | 5 |  |
| Family structure | CZ | Complete family | 307 (30.1) | 402 (39.4) | 214 (21) | 97 (9.5) | 7.63 | 7 | 0.814 (0.135)  0.002 |
|  |  | Incomplete (mother only) | 23 (25) | 47 (51.1) | 14 (15.2) | 8 (8.7) | 7.42 | 6.5 |  |
|  |  | Incomplete (father only) | 5 (21.7) | 7 (30.4) | 9 (39.1) | 2 (8.7) | 8.30 | 8 |  |
|  |  | Divorced parents (with mother) | 57 (22.4) | 117 (45.9) | 48 (18.8) | 33 (12.9) | 8.29 | 7 |  |
|  |  | Divorced parents (with father) | 3 (11.1) | 13 (48.1) | 7 (25.9) | 4 (14.8) | 9.11 | 9 |  |
|  |  | Living with siblings, orphan | 1 (20) | 2 (40) | 2 (40) | (0) | 7.60 | 7 |  |
|  | SK | Complete family | 421 (31) | 573 (42.2) | 265 (19.5) | 100 (7.4) | 7.25 | 7 | 0.295 (0.708)  0.002 |
|  |  | Incomplete (mother only) | 37 (34.6) | 41 (38.3) | 21 (19.6) | 8 (7.5) | 7.27 | 7 |  |
|  |  | Incomplete (father only) | 4 (19) | 10 (47.6) | 6 (28.6) | 1 (4.8) | 7.76 | 7 |  |
|  |  | Divorced parents (with mother) | 43 (25.9) | 76 (45.8) | 33 (19.9) | 14 (8.4) | 7.90 | 7 |  |
|  |  | Divorced parents (with father) | 3 (17.6) | 12 (70.6) | 1 (5.9) | 1 (5.9) | 7.29 | 7 |  |
|  |  | Living with siblings, orphan | 2 (28.6) | 4 (57.1) | 1 (14.3) | (0) | 7.00 | 8 |  |
| Marital status | CZ | Single | 334 (26.7) | 514 (41) | 271 (21.6) | 134 (10.7) | 7.94 | 7 | 16.295 (<0.001)  0.01 |
|  |  | Married | 45 (33.3) | 61 (45.2) | 21 (15.6) | 8 (5.9) | 6.71 | 6 |  |
|  |  | Divorced, widowed | 17 (50) | 13 (38.2) | 2 (5.9) | 2 (5.9) | 5.79 | 4.5 |  |
|  | SK | Single | 468 (29.8) | 673 (42.8) | 314 (20) | 118 (7.5) | 7.38 | 7 | 7.006 (0.030)  0.003 |
|  |  | Married | 38 (38.4) | 43 (43.4) | 13 (13.1) | 5 (5.1) | 6.36 | 6 |  |
|  |  | Divorced, widowed | 4 (80) | (0) | (0) | 1 (20) | 6.40 | 3 |  |

Note: PHQ-15 – Patient Health Questionnaire for somatic symptoms, n – frequency, CZ – Czech Republic, SK – Slovakia

**Supplementary Table 2.** Somatic symptoms (PHQ-15) in the categorization of the study specifics

| PHQ 15 | | | No  n (%) | Mild  n (%) | Moderate  n (%) | Severe  n (%) | Mean | Median | Statistic (p-value)  η^2^ |
| --- | --- | --- | --- | --- | --- | --- | --- | --- | --- |
| Form of study | CZ | Full-time | 268 (25.7) | 437 (42) | 226 (21.7) | 110 (10.6) | 7.98 | 7 | 177231 (0.002)  0.005 |
|  |  | Part-time | 128 (33.6) | 151 (39.6) | 68 (17.8) | 34 (8.9) | 7.20 | 6 |  |
|  | SK | Full-time | 458 (29.5) | 670 (43.2) | 307 (19.8) | 115 (7.4) | 7.38 | 7 | 85434 (0.013)  0.002 |
|  |  | Part-time | 52 (40.9) | 46 (36.2) | 20 (15.7) | 9 (7.1) | 6.61 | 6 |  |
| Degree of study | CZ | 1^st^ degree | 180 (27.4) | 275 (41.8) | 135 (20.5) | 68 (10.3) | 7.69 | 7 | 1.307 (0.727)  0.002 |
|  |  | 2^nd^ degree | 105 (27.6) | 165 (43.4) | 76 (20) | 34 (8.9) | 7.72 | 7 |  |
|  |  | Combined 1^st^ and 2^nd^ degree | 10 (20) | 24 (48) | 7 (14) | 9 (18) | 8.74 | 7 |  |
|  |  | 3^rd^ degree | 101 (30.2) | 124 (37.1) | 76 (22.8) | 33 (9.9) | 7.84 | 7 |  |
|  | SK | 1^st^ degree | 332 (29.1) | 491 (43.1) | 227 (19.9) | 90 (7.9) | 7.42 | 7 | 4-471 (0.215)  0.003 |
|  |  | 2^nd^ degree | 149 (34.8) | 176 (41.1) | 80 (18.7) | 23 (5.4) | 6.95 | 6 |  |
|  |  | Combined 1^st^ and 2^nd^ degree | 10 (24.4) | 16 (39) | 10 (24.4) | 5 (12.2) | 8.32 | 7 |  |
|  |  | 3^rd^ degree | 19 (27.9) | 33 (48.5) | 10 (14.7) | 6 (8.8) | 7.37 | 7 |  |
| Year of study | CZ | 1^st^ | 133 (28.3) | 197 (41.9) | 97 (20.6) | 43 (9.1) | 7.54 | 7 | 7.639 (0.177)  0.008 |
|  |  | 2^nd^ | 127 (29.1) | 178 (40.8) | 88 (20.2) | 43 (9.9) | 7.69 | 7 |  |
|  |  | 3^rd^ | 75 (23.6) | 135 (42.5) | 60 (18.9) | 48 (15.1) | 8.47 | 8 |  |
|  |  | 4^th^ | 25 (26.3) | 39 (41.1) | 29 (30.5) | 2 (2.1) | 7.46 | 7 |  |
|  |  | 5^th^ | 21 (28.8) | 32 (43.8) | 13 (17.8) | 7 (9.6) | 7.63 | 7 |  |
|  |  | 6^th^ | 15 (50) | 7 (23.3) | 7 (23.3) | 1 (3.3) | 6.37 | 5 |  |
|  | SK | 1^st^ | 192 (31.4) | 244 (39.9) | 131 (21.4) | 45 (7.4) | 7.30 | 7 | 11.593 (0.041)  0.008 |
|  |  | 2^nd^ | 152 (29.4) | 230 (44.5) | 88 (17) | 47 (9.1) | 7.47 | 7 |  |
|  |  | 3^rd^ | 71 (24.2) | 134 (45.7) | 63 (21.5) | 25 (8.5) | 7.82 | 7 |  |
|  |  | 4^th^ | 37 (35.6) | 48 (46.2) | 17 (16.3) | 2 (1.9) | 6.41 | 6 |  |
|  |  | 5^th^ | 54 (38.8) | 54 (38.8) | 26 (18.7) | 5 (3.6) | 6.63 | 6 |  |
|  |  | 6^th^ | 4 (33.3) | 6 (50) | 2 (16.7) | (0) | 5.75 | 5 |  |
| Field of study | CZ | Education | 50 (18.1) | 111 (40.1) | 79 (28.5) | 37 (13.4) | 8.92 | 8 | 57.269 (<0.001)  0.04 |
|  |  | Humanities & Arts | 30 (29.7) | 42 (41.6) | 21 (20.8) | 8 (7.9) | 7.44 | 7 |  |
|  |  | Social, Economic & Legal Sciences | 202 (30.4) | 270 (40.6) | 133 (20) | 60 (9) | 7.54 | 7 |  |
|  |  | Natural Science | 14 (28) | 19 (38) | 15 (30) | 2 (4) | 7.56 | 7 |  |
|  |  | Design, Technology, Production & Communications | 44 (47.3) | 37 (39.8) | 7 (7.5) | 5 (5.4) | 5.53 | 5 |  |
|  |  | Agricultural & Veterinary Sciences | 9 (13.4) | 30 (44.8) | 12 (17.9) | 16 (23.9) | 10.03 | 8 |  |
|  |  | Health Service | 12 (22.2) | 26 (48.1) | 5 (9.3) | 11 (20.4) | 8.35 | 7 |  |
|  |  | Services (tourism, sports, security, transport, logistics) | 21 (30.4) | 31 (44.9) | 14 (20.3) | 3 (4.3) | 7.12 | 6 |  |
|  |  | Informatics, Mathematics, ICT | 14 (30.4) | 22 (47.8) | 8 (17.4) | 2 (4.3) | 6.65 | 6.5 |  |
|  | SK | Education | 23 (28.8) | 32 (40) | 15 (18.8) | 10 (12.5) | 7.81 | 7 | 54.91 (<0.001)  0.03 |
|  |  | Humanities & Arts | 13 (16.7) | 34 (43.6) | 20 (25.6) | 11 (14.1) | 8.78 | 9 |  |
|  |  | Social, Economic & Legal Sciences | 202 (30.1) | 299 (44.6) | 132 (19.7) | 38 (5.7) | 7.19 | 7 |  |
|  |  | Natural Science | 23 (31.5) | 37 (50.7) | 12 (16.4) | 1 (1.4) | 6.45 | 6 |  |
|  |  | Design, Technology, Production & Communications | 18 (7.5) | 65 (39.6) | 18 (11) | 8 (4.9) | 5.90 | 5 |  |
|  |  | Agricultural & Veterinary Sciences | 8 (15.1) | 29 (54.7) | 11 (20.8) | 5 (9.4) | 8.53 | 8 |  |
|  |  | Health Service | 38 (21.1) | 70 (38.9) | 50 (27.8) | 22 (12.2) | 8.74 | 8 |  |
|  |  | Services (tourism, sports, security, transport, logistics) | 80 (33.3) | 99 (41.3) | 43 (17.9) | 18 (7.5) | 7.15 | 7 |  |
|  |  | Informatics, Mathematics, ICT | 50 (36.2) | 51 (37) | 26 (18.8) | 11 (8) | 6.94 | 6 |  |

Note: PHQ-15 – Patient Health Questionnaire for somatic symptoms, n – frequency, CZ – Czech Republic, SK – Slovakia, ICT - Information and Communication Technologies

**Supplementary Table 3.** Somatic symptoms (PHQ-15) in the categorization of the residence specifics

| PHQ-15 | | | No  n (%) | Mild  n (%) | Moderate  n (%) | Severe  n (%) | Mean | Median | Statistic  (p-value)  η^2^ |
| --- | --- | --- | --- | --- | --- | --- | --- | --- | --- |
| Distance between home and college | CZ | ≤20.0 | 132 (28.6) | 186 (40.3) | 95 (20.6) | 48 (10.4) | 7.64 | 7 | 0.850 (0.837)  <0.001 |
|  |  | 20.1–50.0 | 91 (28.6) | 121 (38.1) | 76 (23.9) | 30 (9.4) | 7.86 | 8 |  |
|  |  | 50.1–100.0 | 95 (27.2) | 154 (44.1) | 61 (17.5) | 39 (11.2) | 7.82 | 7 |  |
|  |  | ≥100.1 | 78 (26.5) | 127 (43.2) | 62 (21.1) | 27 (9.2) | 7.82 | 7 |  |
|  | SK | ≤20.0 | 131 (32.8) | 165 (41.3) | 79 (19.8) | 25 (6.3) | 7.09 | 6 | 4.360 (0.225)  0.002 |
|  |  | 20.1–50.0 | 103 (28.9) | 161 (45.1) | 76 (21.3) | 17 (4.8) | 7.32 | 7 |  |
|  |  | 50.1–100.0 | 130 (30.7) | 187 (44.1) | 75 (17.7) | 32 (7.5) | 7.17 | 6 |  |
|  |  | ≥100.1 | 145 (29.9) | 197 (40.6) | 94 (19.4) | 49 (10.1) | 7.61 | 7 |  |
| Residence | CZ | Village | 109 (23.9) | 210 (46) | 99 (21.7) | 39 (8.5) | 7.84 | 7 | 0.249 (0.657)  <0.001 |
|  |  | City (up to 10,000) | 68 (26.8) | 100 (39.4) | 67 (26.4) | 19 (7.5) | 7.80 | 7 |  |
|  |  | City (10,001–100,000 | 141 (30.7) | 177 (38.6) | 83 (18.1) | 58 (12.6) | 7.79 | 7 |  |
|  |  | City (100,001–1,000,000) | 51 (30.2) | 69 (40.8) | 34 (20.1) | 15 (8.9) | 7.52 | 7 |  |
|  |  | City (over 1,000,001) | 27 (32.5) | 32 (38.6) | 11 (13.3) | 13 (15.7) | 7.72 | 6 |  |
|  | SK | Village | 243 (29.5) | 354 (43) | 168 (20.4) | 58 (7) | 7.38 | 7 | 3.609 (0.461)  0.003 |
|  |  | City (up to 10,000) | 57 (28.8) | 92 (46.5) | 37 (18.7) | 12 (6.1) | 7.46 | 7 |  |
|  |  | City (10,001–100,000 | 177 (33.7) | 205 (39) | 103 (19.6) | 40 (7.6) | 7.10 | 6 |  |
|  |  | City (100,001–1,000,000) | 30 (25.2) | 62 (52.1) | 15 (12.6) | 12 (10.1) | 7.44 | 6 |  |
|  |  | City (over 1,000,001) | 3 (25) | 3 (25) | 4 (33.3) | 2 (16.7) | 9.67 | 10 |  |
| Housing during the semester | CZ | Dormitory | 54 (22.2) | 105 (43.2) | 58 (23.9) | 26 (10.7) | 8.24 | 8 | 10.211 (0.037)  0.005 |
|  |  | Sublet | 83 (28.9) | 127 (44.3) | 49 (17.1) | 28 (9.8) | 7.59 | 7 |  |
|  |  | With family acquaintances | 53 (26.2) | 86 (42.6) | 39 (19.3) | 24 (11.9) | 7.82 | 7 |  |
|  |  | With a friend | 7 (17.5) | 18 (45) | 9 (22.5) | 6 (15) | 9.18 | 8.5 |  |
|  |  | At home | 199 (30.6) | 252 (38.8) | 139 (21.4) | 60 (9.2) | 7.57 | 7 |  |
|  | SK | Dormitory | 228 (32.5) | 300 (42.7) | 119 (17) | 55 (7.8) | 7.15 | 6 | 23.412 (<0.001)  0.014 |
|  |  | Sublet | 26 (18.7) | 52 (37.4) | 39 (28.1) | 22 (15.8) | 8.97 | 8 |  |
|  |  | With family acquaintances | 17 (25) | 37 (54.4) | 8 (11.8) | 6 (8.8) | 7.66 | 7 |  |
|  |  | With a friend | 5 (16.7) | 14 (46.7) | 8 (26.7) | 3 (10) | 8.60 | 8 |  |
|  |  | At home | 234 (31.7) | 313 (42.4) | 153 (20.7) | 38 (5.1) | 7.09 | 6 |  |

Note: PHQ-15 – Patient Health Questionnaire for somatic symptoms, n – frequency, CZ – Czech Republic, SK – Slovakia

**Supplementary Table 4.** Anxiety (GAD-7) in the categorization of demographic characteristics

| GAD-7 | | | No  n (%) | Mild  n (%) | Moderate  n (%) | Severe  n (%) | Mean | Median | Statistic  (p-value)  η^2^ |
| --- | --- | --- | --- | --- | --- | --- | --- | --- | --- |
| Gender | CZ | Male | 260 (74.5) | 60 (17.2) | 23 (6.6) | 6 (1.7) | 3.32 | 2 | 140009.5 (<0.001)  0.03 |
|  |  | Female | 589 (54.9) | 312 (29.1) | 108 (10.1) | 64 (6) | 5.16 | 4 |  |
|  | SK | Male | 421 (69.5) | 120 (19.8) | 44 (7.3) | 21 (3.5) | 3.74 | 2 | 280983 (<0.001)  0.005 |
|  |  | Female | 676 (63.1) | 265 (24.7) | 92 (8.6) | 38 (3.5) | 4.38 | 3 |  |
| Age | CZ | ≤20 | 99 (51.3) | 55 (28.5) | 20 (10.4) | 19 (9.8) | 5.59 | 4 | 25.276 (<0.001)  0.014 |
|  |  | 21–25 | 524 (58.8) | 243 (27.3) | 87 (9.8) | 37 (4.2) | 4.79 | 4 |  |
|  |  | 26–30 | 110 (64.3) | 38 (22.2) | 12 (7) | 11 (6.4) | 4.49 | 3 |  |
|  |  | ≥31 | 115 (69.3) | 36 (21.7) | 12 (7.2) | 3 (1.8) | 3.46 | 2 |  |
|  | SK | ≤20 | 133 (64.6) | 49 (23.8) | 16 (7.8) | 8 (3.9) | 4.41 | 3 | 15.783 (0.001)  0.004 |
|  |  | 21–25 | 803 (64.8) | 289 (23.3) | 105 (8.5) | 42 (3.4) | 4.19 | 3 |  |
|  |  | 26–30 | 92 (64.3) | 38 (26.6) | 8 (5.6) | 5 (3.5) | 3.94 | 3 |  |
|  |  | ≥31 | 67 (78.8) | 9 (10.6) | 6 (7.1) | 3 (3.5) | 2.99 | 1 |  |
| Family structure | CZ | Complete family | 626 (61.4) | 250 (24.5) | 88 (8.6) | 56 (5.5) | 4.64 | 3 | 5.716 (0.335)  0.002 |
|  |  | Incomplete (mother only) | 57 (62) | 21 (22.8) | 12 (13) | 2 (2.2) | 4.47 | 3 |  |
|  |  | Incomplete (father only) | 11 (47.8) | 9 (39.1) | 2 (8.7) | 1 (4.3) | 4.70 | 5 |  |
|  |  | Divorced parents (with mother) | 140 (54.9) | 79 (31) | 25 (9.8) | 11 (4.3) | 5.05 | 4 |  |
|  |  | Divorced parents (with father) | 13 (48.1) | 11 (40.7) | 3 (11.1) | (0) | 4.81 | 5 |  |
|  |  | Living with siblings, orphan | 2 (40) | 2 (40) | 1 (20) | (0) | 6.00 | 7 |  |
|  | SK | Complete family | 892 (65.6) | 319 (23.5) | 104 (7.7) | 44 (3.2) | 4.09 | 3 | 2.525 (0.773)  0.002 |
|  |  | Incomplete (mother only) | 66 (61.7) | 25 (23.4) | 8 (7.5) | 8 (7.5) | 4.71 | 3 |  |
|  |  | Incomplete (father only) | 14 (66.7) | 3 (14.3) | 2 (9.5) | 2 (9.5) | 4.48 | 3 |  |
|  |  | Divorced parents (with mother) | 111 (66.9) | 34 (20.5) | 16 (9.6) | 5 (3) | 4.03 | 3 |  |
|  |  | Divorced parents (with father) | 11 (64.7) | 2 (11.8) | 4 (23.5) | (0) | 4.71 | 3 |  |
|  |  | Living with siblings, orphan | 3 (42.9) | 2 (28.6) | 2 (28.6) | (0) | 5.86 | 6 |  |
| Marital status | CZ | Single | 733 (58.5) | 333 (26.6) | 120 (9.6) | 67 (5.3) | 4.86 | 4 | 20.377 (<0.001)  0.009 |
|  |  | Married | 89 (65.9) | 34 (25.2) | 10 (7.4) | 2 (1.5) | 3.81 | 3 |  |
|  |  | Divorced, widowed | 27 (79.4) | 5 (14.7) | 1 (2.9) | 1 (2.9) | 2.65 | 1 |  |
|  | SK | Single | 1019 (64.8) | 368 (23.4) | 130 (8.3) | 56 (3.6) | 4.21 | 3 | 9.668 (0.008)  0.004 |
|  |  | Married | 74 (74.7) | 17 (17.2) | 6 (6.1) | 2 (2) | 3.11 | 2 |  |
|  |  | Divorced, widowed | 4 (80) | (0) | (0) | 1 (20) | 3.80 | 0 |  |

Note: GAD-7 – Generalized Anxiety Disorder, n – frequency, CZ – Czech Republic, SK – Slovakia

**Supplementary Table 5.** Anxiety (GAD-7) in the categorization of the study specifics

| GAD-7 | | | No  n (%) | Mild  n (%) | Moderate  n (%) | Severe  n (%) | Mean | Median | Statistic (p-value)  η^2^ |
| --- | --- | --- | --- | --- | --- | --- | --- | --- | --- |
| Form of study | CZ | Full-time | 606 (58.2) | 279 (26.8) | 100 (9.6) | 56 (5.4) | 4.88 | 4 | 178619.5 (0.004)  0.004 |
|  |  | Part-time | 243 (63.8) | 93 (24.4) | 31 (8.1) | 14 (3.7) | 4.24 | 3 |  |
|  | SK | Full-time | 1009 (65.1) | 361 (23.3) | 126 (8.1) | 54 (3.5) | 4.19 | 3 | 87018.5 (0.029)  0.001 |
|  |  | Part-time | 88 (69.3) | 24 (18.9) | 10 (7.9) | 5 (3.9) | 3.65 | 2 |  |
| Degree of study | CZ | 1^st^ degree | 412 (62.6) | 153 (23.3) | 66 (10) | 27 (4.1) | 4.54 | 3 | 4.279 (0.233)  0.003 |
|  |  | 2^nd^ degree | 234 (61.6) | 94 (24.7) | 34 (8.9) | 18 (4.7) | 4.59 | 3 |  |
|  |  | Combined 1^st^ and 2^nd^ degree | 27 (54) | 13 (26) | 3 (6) | 7 (14) | 5.70 | 4 |  |
|  |  | 3^rd^ degree | 176 (52.7) | 112 (33.5) | 28 (8.4) | 18 (5.4) | 5.02 | 4 |  |
|  | SK | 1^st^ degree | 738 (64.7) | 267 (23.4) | 93 (8.2) | 42 (3.7) | 4.23 | 3 | 2.918 (0.404)  0.003 |
|  |  | 2^nd^ degree | 294 (68.7) | 91 (21.3) | 32 (7.5) | 11 (2.6) | 3.82 | 3 |  |
|  |  | Combined 1^st^ and 2^nd^ degree | 28 (68.3) | 6 (14.6) | 4 (9.8) | 3 (7.3) | 4.44 | 2 |  |
|  |  | 3^rd^ degree | 37 (54.4) | 21 (30.9) | 7 (10.3) | 3 (4.4) | 4.69 | 3.5 |  |
| Year of study | CZ | 1^st^ | 287 (61.1) | 116 (24.7) | 41 (8.7) | 26 (5.5) | 4.54 | 3 | 23.377 (<0.001)  0.011 |
|  |  | 2^nd^ | 276 (63.3) | 105 (24.1) | 37 (8.5) | 18 (4.1) | 4.39 | 3 |  |
|  |  | 3^rd^ | 157 (49.4) | 110 (34.6) | 34 (10.7) | 17 (5.3) | 5.55 | 5 |  |
|  |  | 4^th^ | 65 (68.4) | 17 (17.9) | 9 (9.5) | 4 (4.2) | 4.28 | 3 |  |
|  |  | 5^th^ | 43 (58.9) | 19 (26) | 6 (8.2) | 5 (6.8) | 5.00 | 4 |  |
|  |  | 6^th^ | 21 (70) | 5 (16.7) | 4 (13.3) | (0) | 3.60 | 2 |  |
|  | SK | 1^st^ | 400 (65.4) | 141 (23) | 49 (8) | 22 (3.6) | 4.19 | 3 | 7.471 (0.188)  0.007 |
|  |  | 2^nd^ | 334 (64.6) | 129 (25) | 38 (7.4) | 16 (3.1) | 4.03 | 3 |  |
|  |  | 3^rd^ | 175 (59.7) | 72 (24.6) | 32 (10.9) | 14 (4.8) | 4.74 | 3 |  |
|  |  | 4^th^ | 72 (69.2) | 20 (19.2) | 9 (8.7) | 3 (2.9) | 3.81 | 2 |  |
|  |  | 5^th^ | 108 (77.7) | 20 (14.4) | 8 (5.8) | 3 (2.2) | 3.40 | 3 |  |
|  |  | 6^th^ | 8 (66.7) | 3 (25) | (0) | 1 (8.3) | 3.50 | 2 |  |
| Field of study | CZ | Education | 142 (51.3) | 83 (30) | 34 (12.3) | 18 (6.5) | 5.45 | 4 | 32.501 (<0.001)  0.031 |
|  |  | Humanities & Arts | 63 (62.4) | 30 (29.7) | 3 (3) | 5 (5) | 4.29 | 4 |  |
|  |  | Social, Economic & Legal Sciences | 408 (61.4) | 169 (25.4) | 64 (9.6) | 24 (3.6) | 4.48 | 3 |  |
|  |  | Natural Science | 29 (58) | 16 (32) | 5 (10) | (0) | 4.04 | 3 |  |
|  |  | Design, Technology, Production & Communications | 69 (74.2) | 15 (16.1) | 6 (6.5) | 3 (3.2) | 3.55 | 2 |  |
|  |  | Agricultural & Veterinary Sciences | 33 (49.3) | 13 (19.4) | 7 (10.4) | 14 (20.9) | 7.28 | 5 |  |
|  |  | Health Service | 26 (48.1) | 19 (35.2) | 5 (9.3) | 4 (7.4) | 5.54 | 5 |  |
|  |  | Services (tourism, sports, security, transport, logistics) | 45 (65.2) | 17 (24.6) | 5 (7.2) | 2 (2.9) | 4.32 | 4 |  |
|  |  | Informatics, Mathematics, ICT | 34 (73.9) | 10 (21.7) | 2 (4.3) | (0) | 3.28 | 2 |  |
|  | SK | Education | 44 (55) | 16 (20) | 15 (18.8) | 5 (6.3) | 5.30 | 3 | 13.99 (0.082)  0.012 |
|  |  | Humanities & Arts | 48 (61.5) | 18 (23.1) | 9 (11.5) | 3 (3.8) | 4.73 | 4 |  |
|  |  | Social, Economic & Legal Sciences | 460 (68.6) | 154 (23) | 40 (6) | 17 (2.5) | 3.80 | 3 |  |
|  |  | Natural Science | 55 (75.3) | 12 (16.4) | 4 (5.5) | 2 (2.7) | 3.41 | 2 |  |
|  |  | Design, Technology, Production & Communications | 6 (2.5) | 38 (23.2) | 12 (7.3) | 9 (5.5) | 4.20 | 2.5 |  |
|  |  | Agricultural & Veterinary Sciences | 31 (58.5) | 17 (32.1) | 4 (7.5) | 1 (1.9) | 4.23 | 4 |  |
|  |  | Health Service | 107 (59.4) | 48 (26.7) | 16 (8.9) | 9 (5) | 4.72 | 3 |  |
|  |  | Services (tourism, sports, security, transport, logistics) | 162 (67.5) | 47 (19.6) | 25 (10.4) | 6 (2.5) | 3.94 | 3 |  |
|  |  | Informatics, Mathematics, ICT | 85 (61.6) | 35 (25.4) | 11 (8) | 7 (5.1) | 4.71 | 3 |  |

Note: GAD-7 – Generalized Anxiety Disorder, n – frequency, CZ – Czech Republic, SK – Slovakia, ICT - Information and Communication Technologies

**Supplementary Table 6.** Anxiety (GAD-7) in the categorization of the residence specifics

| GAD-7 | | | | No  n (%) | | Mild  n (%) | | Moderate  n (%) | | Severe  n (%) | | Mean | | Median | | Statistic (p-value)  η^2^ |
| --- | --- | --- | --- | --- | --- | --- | --- | --- | --- | --- | --- | --- | --- | --- | --- | --- |
| Distance between home and college | CZ | ≤20.0 | 281 (61) | | 115 (24.9) | | 37 (8) | | 28 (6.1) | | 4.69 | | 3 | | 1.532 (0.675)  <0.001 | |
|  |  | 20.1–50.0 | 184 (57.9) | | 84 (26.4) | | 37 (11.6) | | 13 (4.1) | | 4.70 | | 3 | |  |  |
|  |  | 50.1–100.0 | 210 (60.2) | | 99 (28.4) | | 27 (7.7) | | 13 (3.7) | | 4.70 | | 4 | |  |  |
|  |  | ≥100.1 | 174 (59.2) | | 74 (25.2) | | 30 (10.2) | | 16 (5.4) | | 4.75 | | 3 | |  |  |
|  | SK | ≤20.0 | 274 (68.5) | | 77 (19.3) | | 29 (7.3) | | 20 (5) | | 4.05 | | 3 | | 4.089 (0.252)  0.001 | |
|  |  | 20.1–50.0 | 234 (65.5) | | 89 (24.9) | | 23 (6.4) | | 11 (3.1) | | 4.11 | | 3 | |  |  |
|  |  | 50.1–100.0 | 278 (65.6) | | 98 (23.1) | | 35 (8.3) | | 13 (3.1) | | 4.05 | | 3 | |  |  |
|  |  | ≥100.1 | 307 (63.3) | | 116 (23.9) | | 48 (9.9) | | 14 (2.9) | | 4.28 | | 3 | |  |  |
| Residence | CZ | Village | 273 (59.7) | | 125 (27.4) | | 40 (8.8) | | 19 (4.2) | | 4.63 | | 3 | | 3.679 (0.451)  0.001 | |
|  |  | City (up to 10,000) | 146 (57.5) | | 70 (27.6) | | 24 (9.4) | | 14 (5.5) | | 5.00 | | 4 | |  |  |
|  |  | City (10,001–100,000 | 277 (60.3) | | 113 (24.6) | | 44 (9.6) | | 25 (5.4) | | 4.71 | | 3 | |  |  |
|  |  | City (100,001–1,000,000) | 105 (62.1) | | 42 (24.9) | | 14 (8.3) | | 8 (4.7) | | 4.47 | | 3 | |  |  |
|  |  | City (over 1,000,001) | 48 (57.8) | | 22 (26.5) | | 9 (10.8) | | 4 (4.8) | | 4.73 | | 3 | |  |  |
|  | SK | Village | 522 (63.4) | | 202 (24.5) | | 69 (8.4) | | 30 (3.6) | | 4.31 | | 3 | | 5.786 (0.216)  0.002 | |
|  |  | City (up to 10,000) | 129 (65.2) | | 46 (23.2) | | 16 (8.1) | | 7 (3.5) | | 4.13 | | 3 | |  |  |
|  |  | City (10,001–100,000 | 358 (68.2) | | 109 (20.8) | | 42 (8) | | 16 (3) | | 3.93 | | 2 | |  |  |
|  |  | City (100,001–1,000,000) | 80 (67.2) | | 25 (21) | | 9 (7.6) | | 5 (4.2) | | 3.98 | | 3 | |  |  |
|  |  | City (over 1,000,001) | 8 (66.7) | | 3 (25) | | (0) | | 1 (8.3) | | 4.25 | | 2 | |  |  |
| Housing during the semester | CZ | Dormitory | 140 (57.6) | | 61 (25.1) | | 26 (10.7) | | 16 (6.6) | | 5.14 | | 4 | | 6.865 (0.143)  0.004 | |
|  |  | Sublet | 171 (59.6) | | 79 (27.5) | | 22 (7.7) | | 15 (5.2) | | 4.71 | | 3 | |  |  |
|  |  | With family acquaintances | 110 (54.5) | | 59 (29.2) | | 23 (11.4) | | 10 (5) | | 5.05 | | 4 | |  |  |
|  |  | With a friend | 26 (65) | | 8 (20) | | 2 (5) | | 4 (10) | | 5.12 | | 4 | |  |  |
|  |  | At home | 402 (61.8) | | 165 (25.4) | | 58 (8.9) | | 25 (3.8) | | 4.42 | | 3 | |  |  |
|  | SK | Dormitory | 443 (63.1) | | 182 (25.9) | | 59 (8.4) | | 18 (2.6) | | 4.23 | | 3 | | 8.557 (0.073)  0.004 | |
|  |  | Sublet | 87 (62.6) | | 30 (21.6) | | 13 (9.4) | | 9 (6.5) | | 4.90 | | 3 | |  |  |
|  |  | With family acquaintances | 43 (63.2) | | 17 (25) | | 4 (5.9) | | 4 (5.9) | | 4.26 | | 3 | |  |  |
|  |  | With a friend | 18 (60) | | 10 (33.3) | | 1 (3.3) | | 1 (3.3) | | 4.20 | | 2.5 | |  |  |
|  |  | At home | 506 (68.6) | | 146 (19.8) | | 59 (8) | | 27 (3.7) | | 3.91 | | 2.5 | |  |  |

Note: GAD-7 – Generalized Anxiety Disorder, n – frequency, CZ – Czech Republic, SK – Slovakia

**Supplementary Table 7.** Depression (PHQ-9) in the categorization of demographic characteristics

| PHQ-9 | | | No  n (%) | Mild  n (%) | Moderate  n (%) | Moderately severe  n (%) | Severe  n (%) | Mean | Median | Statistic  (p-value)  η^2^ |
| --- | --- | --- | --- | --- | --- | --- | --- | --- | --- | --- |
| Gender | CZ | Male | 199 (57) | 86 (24.6) | 47 (13.5) | 14 (4) | 3 (0.9) | 5.14 | 4 | 157274 (<0.001)  0.016 |
|  |  | Female | 484 (45.1) | 320 (29.8) | 146 (13.6) | 77 (7.2) | 46 (4.3) | 6.74 | 5 |  |
|  | SK | Male | 329 (54.3) | 169 (27.9) | 59 (9.7) | 30 (5) | 19 (3.1) | 5.58 | 4 | 309466 (0.113)  0.001 |
|  |  | Female | 548 (51.2) | 310 (28.9) | 126 (11.8) | 60 (5.6) | 27 (2.5) | 5.87 | 4 |  |
| Age | CZ | ≤20 | 70 (36.3) | 60 (31.1) | 33 (17.1) | 17 (8.8) | 17 (8.8) | 7.98 | 7 | 49.092 (<0.001)  0.028 |
|  |  | 21–25 | 414 (46.5) | 263 (29.5) | 129 (14.5) | 57 (6.4) | 28 (3.1) | 6.46 | 5 |  |
|  |  | 26–30 | 93 (54.4) | 43 (25.1) | 16 (9.4) | 14 (8.2) | 5 (2.9) | 5.85 | 4 |  |
|  |  | ≥31 | 105 (63.3) | 40 (24.1) | 15 (9) | 3 (1.8) | 3 (1.8) | 4.39 | 3 |  |
|  | SK | ≤20 | 91 (44.2) | 71 (34.5) | 27 (13.1) | 12 (5.8) | 5 (2.4) | 6.26 | 5 | 31.665 (<0.001)  0.009 |
|  |  | 21–25 | 640 (51.7) | 363 (29.3) | 135 (10.9) | 68 (5.5) | 33 (2.7) | 5.83 | 4 |  |
|  |  | 26–30 | 81 (56.6) | 34 (23.8) | 17 (11.9) | 6 (4.2) | 5 (3.5) | 5.64 | 4 |  |
|  |  | ≥31 | 63 (74.1) | 11 (12.9) | 6 (7.1) | 2 (2.4) | 3 (3.5) | 3.75 | 2 |  |
| Family structure | CZ | Complete family | 514 (50.4) | 287 (28.1) | 128 (12.5) | 56 (5.5) | 36 (3.5) | 6.08 | 4 | 10.534 (0.061)  0.007 |
|  |  | Incomplete (mother only) | 38 (41.3) | 31 (33.7) | 14 (15.2) | 6 (6.5) | 3 (3.3) | 6.60 | 5 |  |
|  |  | Incomplete (father only) | 7 (30.4) | 8 (34.8) | 5 (21.7) | 1 (4.3) | 2 (8.7) | 7.91 | 7 |  |
|  |  | Divorced parents (with mother) | 111 (43.5) | 69 (27.1) | 41 (16.1) | 25 (9.8) | 9 (3.5) | 7.10 | 5 |  |
|  |  | Divorced parents (with father) | 10 (37) | 11 (40.7) | 5 (18.5) | 1 (3.7) | (0) | 6.67 | 6 |  |
|  |  | Living with siblings, orphan | 3 (60) | (0) | (0) | 2 (40) | (0) | 8.60 | 4 |  |
|  | SK | Complete family | 712 (52.4) | 397 (29.2) | 148 (10.9) | 72 (5.3) | 30 (2.2) | 5.63 | 4 | 4.075 (0.539)  0.005 |
|  |  | Incomplete (mother only) | 57 (53.3) | 21 (19.6) | 15 (14) | 9 (8.4) | 5 (4.7) | 6.94 | 4 |  |
|  |  | Incomplete (father only) | 11 (52.4) | 6 (28.6) | 2 (9.5) | 1 (4.8) | 1 (4.8) | 5.95 | 4 |  |
|  |  | Divorced parents (with mother) | 86 (51.8) | 46 (27.7) | 17 (10.2) | 8 (4.8) | 9 (5.4) | 6.13 | 4 |  |
|  |  | Divorced parents (with father) | 8 (47.1) | 7 (41.2) | 2 (11.8) | (0) | (0) | 5.12 | 5 |  |
|  |  | Living with siblings, orphan | 3 (42.9) | 2 (28.6) | 1 (14.3) | (0) | 1 (14.3) | 7.86 | 7 |  |
| Marital status | CZ | Single | 586 (46.8) | 359 (28.7) | 179 (14.3) | 82 (6.5) | 47 (3.8) | 6.53 | 5 | 21.396 (<0.001)  0.01 |
|  |  | Married | 72 (53.3) | 42 (31.1) | 12 (8.9) | 8 (5.9) | 1 (0.7) | 5.34 | 4 |  |
|  |  | Divorced, widowed | 25 (73.5) | 5 (14.7) | 2 (5.9) | 1 (2.9) | 1 (2.9) | 3.65 | 1 |  |
|  | SK | Single | 800 (50.9) | 465 (29.6) | 178 (11.3) | 89 (5.7) | 41 (2.6) | 5.88 | 4 | 26.749 (0.001)  0.008 |
|  |  | Married | 73 (73.7) | 14 (14.1) | 6 (6.1) | 1 (1) | 5 (5.1) | 4.03 | 2 |  |
|  |  | Divorced, widowed | 4 (80) | (0) | 1 (20) | (0) | (0) | 3.20 | 0 |  |

Note: PHQ-9 – Patient Health Questionnaire for depression, n – frequency, CZ – Czech Republic, SK – Slovakia

**Supplementary Table 8.** Depression (PHQ-9) in the categorization of the study specifics

| PHQ-9 | | | No  n (%) | Mild  n (%) | Moderate  n (%) | Moderately severe  n (%) | Severe  n (%) | Mean | Median | Statistic  (p-value)  η^2^ |
| --- | --- | --- | --- | --- | --- | --- | --- | --- | --- | --- |
| Form of study | CZ | Full-time | 474 (45.5) | 299 (28.7) | 154 (14.8) | 73 (7) | 41 (3.9) | 6.72 | 5 | 166294.5 (<0.001)  0.013 |
|  |  | Part-time | 209 (54.9) | 107 (28.1) | 39 (10.2) | 18 (4.7) | 8 (2.1) | 5.31 | 4 |  |
|  | SK | Full-time | 793 (51.2) | 455 (29.4) | 176 (11.4) | 84 (5.4) | 42 (2.7) | 5.86 | 4 | 78542 (<0.001)  0.004 |
|  |  | Part-time | 84 (66.1) | 24 (18.9) | 9 (7.1) | 6 (4.7) | 4 (3.1) | 4.61 | 3 |  |
| Degree of study | CZ | 1^st^ degree | 317 (48.2) | 193 (29.3) | 90 (13.7) | 37 (5.6) | 21 (3.2) | 6.26 | 5 | 8.607 (0.035)  0.009 |
|  |  | 2^nd^ degree | 199 (52.4) | 105 (27.6) | 41 (10.8) | 27 (7.1) | 8 (2.1) | 5.90 | 4 |  |
|  |  | Combined 1^st^ and 2^nd^ degree | 17 (34) | 13 (26) | 7 (14) | 8 (16) | 5 (10) | 8.68 | 7 |  |
|  |  | 3^rd^ degree | 150 (44.9) | 95 (28.4) | 55 (16.5) | 19 (5.7) | 15 (4.5) | 6.67 | 5 |  |
|  | SK | 1^st^ degree | 578 (50.7) | 341 (29.9) | 121 (10.6) | 67 (5.9) | 33 (2.9) | 5.88 | 4 | 2.45 (0.484)  0.002 |
|  |  | 2^nd^ degree | 243 (56.8) | 112 (26.2) | 47 (11) | 17 (4) | 9 (2.1) | 5.42 | 4 |  |
|  |  | Combined 1^st^ and 2^nd^ degree | 25 (61) | 4 (9.8) | 9 (22) | 3 (7.3) | (0) | 5.83 | 3 |  |
|  |  | 3^rd^ degree | 31 (45.6) | 22 (32.4) | 8 (11.8) | 3 (4.4) | 4 (5.9) | 6.04 | 5 |  |
| Year of study | CZ | 1^st^ | 221 (47) | 145 (30.9) | 53 (11.3) | 30 (6.4) | 21 (4.5) | 6.33 | 5 | 11.779 (0.038)  0.007 |
|  |  | 2^nd^ | 232 (53.2) | 112 (25.7) | 54 (12.4) | 25 (5.7) | 13 (3) | 5.98 | 4 |  |
|  |  | 3^rd^ | 129 (40.6) | 94 (29.6) | 60 (18.9) | 26 (8.2) | 9 (2.8) | 7.11 | 6 |  |
|  |  | 4^th^ | 50 (52.6) | 23 (24.2) | 13 (13.7) | 7 (7.4) | 2 (2.1) | 5.98 | 4 |  |
|  |  | 5^th^ | 35 (47.9) | 24 (32.9) | 9 (12.3) | 2 (2.7) | 3 (4.1) | 6.08 | 5 |  |
|  |  | 6^th^ | 16 (53.3) | 8 (26.7) | 4 (13.3) | 1 (3.3) | 1 (3.3) | 5.50 | 4 |  |
|  | SK | 1^st^ | 313 (51.1) | 188 (30.7) | 63 (10.3) | 31 (5.1) | 17 (2.8) | 5.78 | 4 | 9.509 (0.09)  0.007 |
|  |  | 2^nd^ | 279 (54) | 141 (27.3) | 57 (11) | 29 (5.6) | 11 (2.1) | 5.55 | 4 |  |
|  |  | 3^rd^ | 136 (46.4) | 86 (29.4) | 37 (12.6) | 21 (7.2) | 13 (4.4) | 6.66 | 5 |  |
|  |  | 4^th^ | 58 (55.8) | 27 (26) | 12 (11.5) | 5 (4.8) | 2 (1.9) | 5.31 | 4 |  |
|  |  | 5^th^ | 83 (59.7) | 34 (24.5) | 16 (11.5) | 4 (2.9) | 2 (1.4) | 5.12 | 4 |  |
|  |  | 6^th^ | 8 (66.7) | 3 (25) | (0) | (0) | 1 (8.3) | 4.67 | 3.5 |  |
| Field of study | CZ | Education | 112 (40.4) | 85 (30.7) | 40 (14.4) | 24 (8.7) | 16 (5.8) | 7.27 | 6 | 36.628 (<0.001)  0.034 |
|  |  | Humanities & Arts | 56 (55.4) | 26 (25.7) | 13 (12.9) | 4 (4) | 2 (2) | 5.61 | 4 |  |
|  |  | Social, Economic & Legal Sciences | 337 (50.7) | 186 (28) | 87 (13.1) | 41 (6.2) | 14 (2.1) | 5.92 | 4 |  |
|  |  | Natural Science | 26 (52) | 13 (26) | 9 (18) | 2 (4) | (0) | 5.72 | 4 |  |
|  |  | Design, Technology, Production & Communications | 51 (54.8) | 25 (26.9) | 10 (10.8) | 4 (4.3) | 3 (3.2) | 5.31 | 3 |  |
|  |  | Agricultural & Veterinary Sciences | 23 (34.3) | 14 (20.9) | 12 (17.9) | 10 (14.9) | 8 (11.9) | 9.84 | 9 |  |
|  |  | Health Service | 21 (38.9) | 22 (40.7) | 6 (11.1) | 1 (1.9) | 4 (7.4) | 7.20 | 6 |  |
|  |  | Services (tourism, sports, security, transport, logistics) | 36 (52.2) | 22 (31.9) | 7 (10.1) | 4 (5.8) | (0) | 5.39 | 4 |  |
|  |  | Informatics, Mathematics, ICT | 21 (45.7) | 13 (28.3) | 9 (19.6) | 1 (2.2) | 2 (4.3) | 6.65 | 5 |  |
|  | SK | Education | 41 (51.3) | 22 (27.5) | 8 (10) | 3 (3.8) | 6 (7.5) | 6.19 | 4 | 18.384 (0.019)  0.011 |
|  |  | Humanities & Arts | 31 (39.7) | 26 (33.3) | 12 (15.4) | 5 (6.4) | 4 (5.1) | 7.01 | 5.5 |  |
|  |  | Social, Economic & Legal Sciences | 377 (56.2) | 188 (28) | 61 (9.1) | 32 (4.8) | 13 (1.9) | 5.28 | 4 |  |
|  |  | Natural Science | 40 (54.8) | 19 (26) | 9 (12.3) | 3 (4.1) | 2 (2.7) | 5.62 | 4 |  |
|  |  | Design, Technology, Production & Communications | 11 (4.6) | 49 (29.9) | 18 (11) | 7 (4.3) | 8 (4.9) | 6.18 | 4.5 |  |
|  |  | Agricultural & Veterinary Sciences | 24 (45.3) | 14 (26.4) | 11 (20.8) | 3 (5.7) | 1 (1.9) | 6.77 | 6 |  |
|  |  | Health Service | 90 (50) | 46 (25.6) | 28 (15.6) | 14 (7.8) | 2 (1.1) | 6.06 | 4.5 |  |
|  |  | Services (tourism, sports, security, transport, logistics) | 135 (56.3) | 63 (26.3) | 26 (10.8) | 11 (4.6) | 5 (2.1) | 5.43 | 4 |  |
|  |  | Informatics, Mathematics, ICT | 57 (41.3) | 52 (37.7) | 12 (8.7) | 12 (8.7) | 5 (3.6) | 6.58 | 5 |  |

Note: PHQ-9 – Patient Health Questionnaire for depression, n – frequency, CZ – Czech Republic, SK – Slovakia, ICT - Information and Communication Technologies

**Supplementary Table 9.** Depression (PHQ-9) in the categorization of the residence specifics

| PHQ-9 | | | No  n (%) | Mild  n (%) | Moderate  n (%) | Moderately severe  n (%) | Severe  n (%) | Mean | Median | Statistic  (p-value)  η^2^ |
| --- | --- | --- | --- | --- | --- | --- | --- | --- | --- | --- |
| Distance between home and college | CZ | ≤20.0 | 229 (49.7) | 129 (28) | 58 (12.6) | 25 (5.4) | 20 (4.3) | 6.26 | 5 | 1.807 (0.613)  0.001 |
|  |  | 20.1–50.0 | 156 (49.1) | 87 (27.4) | 46 (14.5) | 20 (6.3) | 9 (2.8) | 6.19 | 5 |  |
|  |  | 50.1–100.0 | 162 (46.4) | 110 (31.5) | 45 (12.9) | 22 (6.3) | 10 (2.9) | 6.27 | 5 |  |
|  |  | ≥100.1 | 136 (46.3) | 80 (27.2) | 44 (15) | 24 (8.2) | 10 (3.4) | 6.73 | 5 |  |
|  | SK | ≤20.0 | 206 (51.5) | 117 (29.3) | 43 (10.8) | 21 (5.3) | 13 (3.3) | 5.73 | 4 | 9.573 (0.023)  0.003 |
|  |  | 20.1–50.0 | 183 (51.3) | 108 (30.3) | 36 (10.1) | 22 (6.2) | 8 (2.2) | 5.73 | 4 |  |
|  |  | 50.1–100.0 | 240 (56.6) | 116 (27.4) | 36 (8.5) | 20 (4.7) | 12 (2.8) | 5.36 | 4 |  |
|  |  | ≥100.1 | 242 (49.9) | 137 (28.2) | 67 (13.8) | 27 (5.6) | 12 (2.5) | 6.15 | 5 |  |
| Residence | CZ | Village | 224 (49) | 132 (28.9) | 60 (13.1) | 28 (6.1) | 13 (2.8) | 6.21 | 5 | 1.264 (0.868)  0.001 |
|  |  | City (up to 10,000) | 112 (44.1) | 82 (32.3) | 35 (13.8) | 18 (7.1) | 7 (2.8) | 6.58 | 5 |  |
|  |  | City (10,001–100,000 | 223 (48.6) | 121 (26.4) | 66 (14.4) | 28 (6.1) | 21 (4.6) | 6.36 | 5 |  |
|  |  | City (100,001–1,000,000) | 82 (48.5) | 53 (31.4) | 20 (11.8) | 11 (6.5) | 3 (1.8) | 6.08 | 5 |  |
|  |  | City (over 1,000,001) | 42 (50.6) | 18 (21.7) | 12 (14.5) | 6 (7.2) | 5 (6) | 6.82 | 4 |  |
|  | SK | Village | 437 (53.1) | 233 (28.3) | 93 (11.3) | 45 (5.5) | 15 (1.8) | 5.63 | 4 | 0.977 (0.913)  <0.001 |
|  |  | City (up to 10,000) | 98 (49.5) | 56 (28.3) | 23 (11.6) | 13 (6.6) | 8 (4) | 6.11 | 5 |  |
|  |  | City (10,001–100,000 | 271 (51.6) | 156 (29.7) | 56 (10.7) | 23 (4.4) | 19 (3.6) | 5.85 | 4 |  |
|  |  | City (100,001–1,000,000) | 63 (52.9) | 32 (26.9) | 13 (10.9) | 8 (6.7) | 3 (2.5) | 5.81 | 4 |  |
|  |  | City (over 1,000,001) | 8 (66.7) | 2 (16.7) | (0) | 1 (8.3) | 1 (8.3) | 5.50 | 3 |  |
| Housing during the semester | CZ | Dormitory | 95 (39.1) | 71 (29.2) | 53 (21.8) | 18 (7.4) | 6 (2.5) | 7.23 | 6 | 19.867 (0.001)  0.008 |
|  |  | Sublet | 143 (49.8) | 80 (27.9) | 34 (11.8) | 17 (5.9) | 13 (4.5) | 6.38 | 5 |  |
|  |  | With family acquaintances | 92 (45.5) | 64 (31.7) | 24 (11.9) | 13 (6.4) | 9 (4.5) | 6.60 | 5 |  |
|  |  | With a friend | 20 (50) | 11 (27.5) | 3 (7.5) | 2 (5) | 4 (10) | 6.65 | 5 |  |
|  |  | At home | 333 (51.2) | 180 (27.7) | 79 (12.2) | 41 (6.3) | 17 (2.6) | 5.90 | 4 |  |
|  | SK | Dormitory | 357 (50.9) | 204 (29.1) | 84 (12) | 38 (5.4) | 19 (2.7) | 5.92 | 4 | 11.825 (0.019)  0.007 |
|  |  | Sublet | 66 (47.5) | 36 (25.9) | 21 (15.1) | 9 (6.5) | 7 (5) | 6.76 | 5 |  |
|  |  | With family acquaintances | 33 (48.5) | 17 (25) | 10 (14.7) | 4 (5.9) | 4 (5.9) | 6.63 | 5 |  |
|  |  | With a friend | 15 (50) | 7 (23.3) | 5 (16.7) | 2 (6.7) | 1 (3.3) | 6.13 | 4.5 |  |
|  |  | At home | 406 (55) | 215 (29.1) | 65 (8.8) | 37 (5) | 15 (2) | 5.34 | 4 |  |

Note: PHQ-9 – Patient Health Questionnaire for depression, n – frequency, CZ – Czech Republic, SK – Slovakia
